# Atomistic Simulations of Fe(CO)_5_ Fragmentation Dynamics on a Substrate

**DOI:** 10.1021/acs.jpcc.5c08601

**Published:** 2026-06-04

**Authors:** Hlib Lyshchuk, Alexey V. Verkhovtsev, Juraj Fedor, Andrey V. Solov’yov

**Affiliations:** † 86875 J. Heyrovský Institute of Physical Chemistry, Czech Academy of Sciences, Dolejškova 3, 18223 Prague, Czech Republic; ‡ Department of Physical Chemistry, University of Chemistry and Technology, Technická 5, 16628 Prague, Czech Republic; ¶ MBN Research Center, Altenhöferallee 3, 60438 Frankfurt am Main, Germany

## Abstract

The dissociation of organometallic molecules upon ionization by electron impact is one of the key fundamental processes behind nanofabrication using focused electron beams. The degree of molecular fragmentation (i.e., the number of volatile ligands produced) and the dynamics of this process are important factors affecting the elemental composition and metal content of the resulting deposits. This study presents results of irradiation-driven molecular dynamics (IDMD) simulations of the irradiation-induced fragmentation of iron pentacarbonyl, Fe­(CO)_5_, physisorbed on several different experimentally relevant substrates: crystalline Au(111) and Au(100), polycrystalline gold, and amorphous carbon. The employed computational approach enables the simulation of covalent bond breaking in the deposited molecule alongside the explicit dynamics of the substrate. We demonstrate that the fragmentation dynamics of Fe­(CO)_5_ is similar across the considered substrates but is influenced by the strength of its binding to the substrate and by the structural irregularity of the substrate, which enables the molecule to adopt various orientations. The results of this study have implications for atomistic simulations of nanostructure formation using focused electron beams and for the interpretation of surface-science experiments involving the irradiation of molecular systems with electron beams.

## Graphical abstract

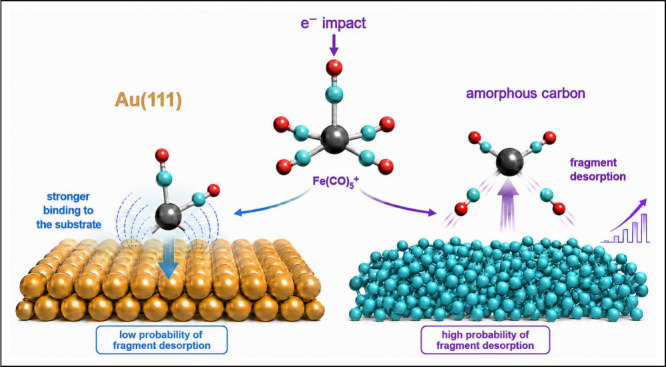

## Introduction

Focused electron beam-induced deposition (FEBID)^1–4^ is a modern “bottom-up” nanofabrication technique that exploits the spatially localized irradiation-driven chemistry of organometallic precursor molecules adsorbed onto a surface.^5,6^ When the molecules are exposed to collimated beams of charged particles, they undergo chemical transformations, leading to the formation and growth of metal-containing deposits. This enables direct-write 3D nanoprinting of complex structures with a spatial resolution of several tens of nanometers.^7–9^ A significant technological challenge for this approach is fabricating metal nanostructures with the desired geometry, size and purity. Incomplete separation of organic ligands from metal atoms often results in nanostructures with low metal content and contamination by unwanted atoms such as carbon and oxygen. Consequently, considerable effort has been devoted in recent years to improving our understanding of the electron-induced chemistry and fragmentation mechanisms of FEBID precursors.^4,6^

Alongside experimental developments in 3D nanoprinting using focused charged particle beams, significant advances have been made over the past decade in computational multiscale modeling of the FEBID process.^10–16^ These studies have successfully demonstrated the effectiveness of a multiscale modeling approach in simulating the formation and growth of metal-containing deposits during FEBID, and in providing a quantitative description of their key characteristics, such as composition, size and growth rate. The developed approach encompasses the entire set of FEBID-related processes occurring over different time and spatial scales, including (i) the interaction of primary radiation and secondary electrons with the target systems; (ii) radiation-induced chemical reactions (molecular fragmentation and association); (iii) molecular diffusion, adsorption, desorption, and aggregation; and (iv) postirradiation phenomena, such as structural and morphological transformations.

The detailed atomistic description of the radiation-driven chemical processes underlying FEBID has become possible using the advanced irradiation-driven molecular dynamics (IDMD) method formulated in ref 11. IDMD has been used for atomistic simulations of radiation-induced chemistry involving FEBID precursors^17–19^ and for the atomistic-level characterization of nanostructures grown using FEBID, including the prediction of their morphology, growth rate, and geometrical (e.g., lateral size, height, and volume) and chemical (metal content) characteristics.^11–15^ A relevant scientific question concerns the elucidation of the mechanisms of radiation-driven fragmentation of deposited precursors and their interaction with the substrate, including the quantitative description of the energy transfer and dissipation processes between the deposited precursors exposed to irradiation and the substrate. A molecular-level understanding of the surface chemistry involved in the FEBID process still remains incomplete and is the subject of active investigation.^20,21^

Different groups of organometallic precursors for FEBID have been studied, as discussed in detail in the recent reviews.^4,6^ One of the most widely used FEBID precursors is iron pentacarbonyl, Fe­(CO)_5_. This molecule has attracted significant interest from the FEBID community due to its high volatility, the ability to produce deposits with a high metal content, and well-characterized routines for fabricating iron-based nanostructures with specific magnetic properties for use in magnetic sensing, spintronics, and magnetologic technologies.^22–27^

The use of iron pentacarbonyl in FEBID has prompted extensive fundamental research into electron interactions with this molecule^28–33^ and its derivatives, such as Fe­(CO)_4_(methyl acrylate).^34–37^ A key focus of these studies has been analyzing the fragmentation pattern to determine how many carbonyl ligands are cleaved from the iron atom. This has been investigated for molecules in the gas phase^28–30^ and in the condensed phase, considering thin Fe­(CO)_5_ films adsorbed on surfaces.^31–33^ A notable effect has been observed in cluster beam experiments, which allow gas-phase techniques to be applied to aggregated precursors.^38–40^ It has been found^38^ that fragmentation of Fe­(CO)_5_ due to electron impact ionization (i.e., in the dissociative ionization process) is strongly suppressed upon clusterization. This was later explained by our previous MD simulations^18^ using the reactive force fields,^41^ which compared the fragmentation of a gas-phase Fe­(CO)_5_ molecule with that embedded in an argon cluster. These simulations showed that, in an argon environment, the Fe­(CO)_5_^+^ cation should possess a much larger internal energy than an isolated cation in order to have a comparable degree of fragmentation.

These previous results naturally raise the question of how the molecular fragmentation due to dissociative ionization is affected when precursor molecules are physisorbed onto a substrate. It is reasonable to expect the substrate to quench the internal energy of the parent cation and influence its dissociation. In order to provide a detailed characterization of this process, we present here the results of IDMD simulations of the radiation-induced fragmentation of the Fe­(CO)_5_^+^ ion physisorbed on several different substrates: crystalline Au(111) and Au(100), polycrystalline gold, and amorphous carbon (a-C) – while explicitly considering the dynamics of the substrates. The substrates studied here have been commonly used in surface science experiments involving organometallic precursors for FEBID.^20,33,42–47^ In the recent work of Barnewitz et al.,^37^ it was pointed out that the research related to FEBID has so far not paid much attention to the nature of precursor interactions with deposits. The choice of substrates in this study reflects two limiting physical scenarios that are relevant to 3D nanoprinting conditions. In this process, the precursors initially dissociate on a clean, even surface. As the deposit grows, freshly delivered precursor molecules are physisorbed and dissociate on the deposited material, which is often uneven/less structured and has a high carbon content.

## Computational Methodology

IDMD simulations of radiation-induced fragmentation of physisorbed Fe­(CO)_5_^+^ were performed using MBN Explorer,^48^ a software package for multiscale simulations of the structure and dynamics of complex meso-bio-nano (MBN) systems.^49^ The MBN Studio toolkit^50^ was used to create the system’s structure, prepare the necessary input files, and analyze the simulation results.

The interatomic interactions for Fe­(CO)_5_^+^ and its fragments were described using the reactive CHARMM (rCHARMM) force field^41^ implemented in MBN Explorer. It allows the simulation of various molecular systems with the dynamically changing molecular topology, which is essential for modeling chemical transformations in various molecular and condensed matter systems, including those induced by irradiation.^10,12–15,17,19,51–53^ In contrast to the “standard” (nonreactive) CHARMM force field,^54,55^ which was developed for simulating the structure and near-equilibrium dynamics of biomolecular systems, the rCHARMM force field^41^ is more versatile and can be used to simulate various molecular systems, including biomolecular,^52,53^ inorganic,^51^ and organometallic systems, including FEBID precursors studied in this work and in a number of previous studies.^11–15,17,19^

To permit the rupture and formation of covalent bonds, the radial part of bonded interactions is described in rCHARMM using a Morse potential instead of a harmonic potential as in the “standard” CHARMM force fields.^54^ The rupture of covalent bonds in the simulation automatically implies a modification of the potential energy functions for angular interactions;^41^ see Supporting Information for further details. The parameters for bonded and angular interactions, nonbonded interactions, and atomic partial charges in the parent Fe­(CO)_5_^+^ ion and its molecular fragments are listed in Tables S3–S6.

All parameters except those for nonbonded interactions were calculated in this study using density functional theory (DFT) with the Gaussian 16 software package.^56^ The calculations employed the hybrid B3LYP functional,^57^ the 6–31+G­(d) basis set,^58,59^ and the GD3 empirical dispersion correction.^60^ Further computational details and relevant benchmarks are provided in the Supporting Information (see Tables S1 and S2).

The methodology for simulating the radiation-induced fragmentation of Fe­(CO)_5_^+^ is based on the previous studies of the radiation-induced fragmentation of several isolated organometallic molecules.^17–19^ The key difference is that the present simulations have been performed in the presence of a substrate whose dynamics has been treated explicitly. As the main objects of our study, we have considered an Au(111) substrate with the size of 99.69 Å × 100.73 Å, containing 4200 atoms, and an amorphous carbon (a-C) substrate with the size of 89.17 Å × 89.17 Å, containing 8690 atoms (see Figure 1). To study the impact of surface orientation on the fragmentation dynamics of Fe­(CO)_5_^+^, we also considered two alternative gold substrates: Au(100) containing 5000 atoms and polycrystalline gold (containing 5618 atoms), which consists of multiple randomly oriented grains (see Figure S3). The in-plane size of both substrates was 101.96 Å × 101.96 Å. The corresponding results are presented in the Supporting Information (Figures S4–S6). The detailed computational procedure for constructing the initial geometries of all substrates is also described in the Supporting Information.

**Figure 1:**
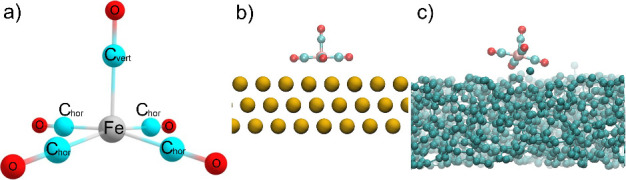
(a) Optimized geometry of a Fe­(CO)_5_^+^ cation considered in this study: a square-pyramidal *C*_4*v*_ isomer.^29,61,62^ Different atom types are indicated. The “vertical” Fe–C_vert_ bond is almost orthogonal to the four “horizontal” Fe–C_hor_ bonds, with the C_hor_–Fe–C_vert_ angle being equal to ∼97°. Panels (b) and (c) show the optimized configurations of Fe­(CO)_5_^+^ deposited on the Au(111) substrate (panel (b)) and an amorphous carbon (a-C) substrate (panel (c)).

Regarding the simulations of Fe­(CO)_5_^+^ fragmentation, the geometry of the Fe­(CO)_5_^+^ cation was first optimized on each substrate using MBN Explorer, with the interaction parameters listed in Tables S3–S6. The lowest-energy isomer of the Fe­(CO)_5_^+^ cation has a square-pyramidal *C*_4*v*_ structure,^29,61,62^ as shown in Figure 1a. This was confirmed by DFT calculations for a free Fe­(CO)_5_^+^ cation and for a cation placed on flat gold and carbon nanosupports (see Figure S1 as well as Tables S1 and S2).

The interatomic interactions in the gold substrates were described using the many-body Gupta potential^63^ with the parameters taken from ref 64. We considered an ideal Au(111) surface and did not account for surface reconstruction,^65^ which occurs due to the closer packing of gold atoms in the Au(111) surface atomic layer compared to the bulk. Simulating these effects would require the use of more sophisticated interatomic potentials for gold.^65,66^ In contrast, the approach used here has been successfully used recently to study the structural and dynamic properties of self-assembled monolayers of alkanethiols on an Au(111) substrate.^67^ The interactions between carbon atoms in the a-C substrate were described using the many-body Brenner potential.^68^

After geometry optimization, each system was thermalized at *T* = 300 K for 200 ps. The MD simulations were carried out using a Langevin thermostat with a damping time of 0.2 ps. Twelve independent simulations were performed for each substrate for statistical purposes. In each trajectory, atomic coordinates and velocities for all atoms in the system were recorded every 10 ps. These trajectories were subsequently used to generate initial geometries and velocity distributions for the follow-up IDMD simulations of radiation-induced Fe­(CO)_5_^+^ fragmentation.

The IDMD simulations of radiation-induced Fe­(CO)_5_^+^ fragmentation were performed in a simulation box with the following dimensions: 99.69 × 100.73 × 200 Å^3^ for Au(111), 101.96 × 101.96 × 200 Å^3^ for Au(100) and polycrystalline gold, and 89.17 × 89.17 × 200 Å^3^ for the a-C substrate. All simulations were run for 0.5 ns with an integration time step of 0.1 fs and without a thermostat. The relatively small time step was chosen to ensure consistency and stability in all the simulations performed, including those involving the transfer of large amounts of energy to the deposited molecule. A total of 1,860 simulations were carried out for each of the Au(111) and a-C substrates, and a 2-fold smaller number of simulations for each of the Au(100) and polycrystalline gold substrates. Different values of the excess energy were deposited into the Fe­(CO)_5_^+^, ranging from 0 to approximately ∼21.7 eV (500 kcal/mol). The excess energy was varied in 0.43 eV (10 kcal/mol) increments from 0 to 8.67 eV (0 to 200 kcal/mol), in 0.87 eV (20 kcal/mol) increments from 8.67 to 13.01 eV (200 to 300 kcal/mol), and in 2.17 eV (50 kcal/mol) increments from 13.01 to 21.68 eV (300 to 500 kcal/mol). For each energy value, 60 independent simulations were performed. The molecular fragments produced at the end of each simulation were analyzed, and the corresponding fragment appearance energies (AEs) and their abundances were determined from this analysis.

Each simulation run corresponds to the unimolecular fragmentation of the Fe­(CO)_5_^+^ cation. The methodology used in this study was previously described in detail^17–19^ and is briefly summarized here for clarity. At simulation time zero, the molecular cation is assumed to be in its electronic ground state, with a specified amount of excess energy distributed among its vibrational degrees of freedom. This model reflects the physical scenario that, upon the ionization of the neutral molecule by an incoming electron, the internal energy is transferred from the electronic to the nuclear degrees of freedom on an ultrafast time scale. An explicit treatment of quantum nonadiabatic dynamics show that although electrons are removed from a broad range of molecular orbitals during ionization—producing cations in many electronically excited states—these excited states decay nonadiabatically to the ground state on a femto- to picosecond time scale.^69^ The excess energy remaining in the cation after ionization is therefore transferred into vibrational energy, which serves as the starting point for classical IDMD simulations.

The IDMD methodology allows the analysis of fast energy transfer events into fragmenting covalent bonds caused by radiation-induced quantum processes (e.g., dissociative ionization considered in this study), as well as the analysis of postirradiation energy relaxation processes that typically occur on the picosecond time scale and lead to chemical transformations.^10,11,17–19^ In the fast and localized energy transfer mechanism, the energy remaining in the system after ionization (i.e., the excess energy over the ionization energy of the parent molecule) is locally transferred to a specific covalent bond of the parent ion and converted into the kinetic energy of the two atoms forming the bond. In the slow thermal energy transfer mechanism, the energy stored in the electronic degrees of freedom is statistically distributed to the vibrational degrees of freedom of the parent ion. The equilibrium atomic velocities corresponding to a given temperature (300 K in the present simulations) have been scaled depending on the amount of energy transferred to the system.

Three distinct energy deposition scenarios were considered: (i) the uniform energy distribution over all atoms due to the thermal energy transfer mechanism; (ii) a localized energy transfer into a Fe–C_vert_ bond (see atomic notations in Figure 1a); and (iii) localized energy deposition into one of Fe–C_hor_ bonds (see Figure 1a). These energy deposition scenarios capture different physical mechanisms of internal energy transfer from the electronic to nuclear degrees of freedom, as described above. After the initial energy deposition, classical MD simulations were carried out, explicitly taking into account the dynamics of the substrate. At the end of each simulated trajectory, the created molecular fragments were analyzed.

For statistical purposes, 60 independent initial configurations of Fe­(CO)_5_^+^ were sampled for each substrate, reflecting the configurational variability expected in experiments. The results of the performed simulations reveal how variations in the substrate geometry, orientation of the molecule on the substrate, and the binding energy between the molecule and the substrate influence the fragmentation pathways and desorption behavior of Fe-containing species.

## Results and Discussion

### Structural and Energetic Characteristics of the Physisorbed Fe­(CO)_5_^+^

We begin this section by analyzing the structural characteristics and energetics of the “Fe­(CO)_5_^+^–substrate” system prior to the deposition of the excess energy into the parent cation. Figure 2 shows the results of the statistical analysis for different Fe­(CO)_5_^+^ adsorption geometries on Au(111) and a-C substrates, obtained from the performed MD simulations. A similar comparison of the structural characteristics and energetics of Fe­(CO)_5_^+^ physisorbed on Au(111), Au(100) and polycrystalline gold substrates is given in the Supporting Information (Figure S4).

**Figure 2:**
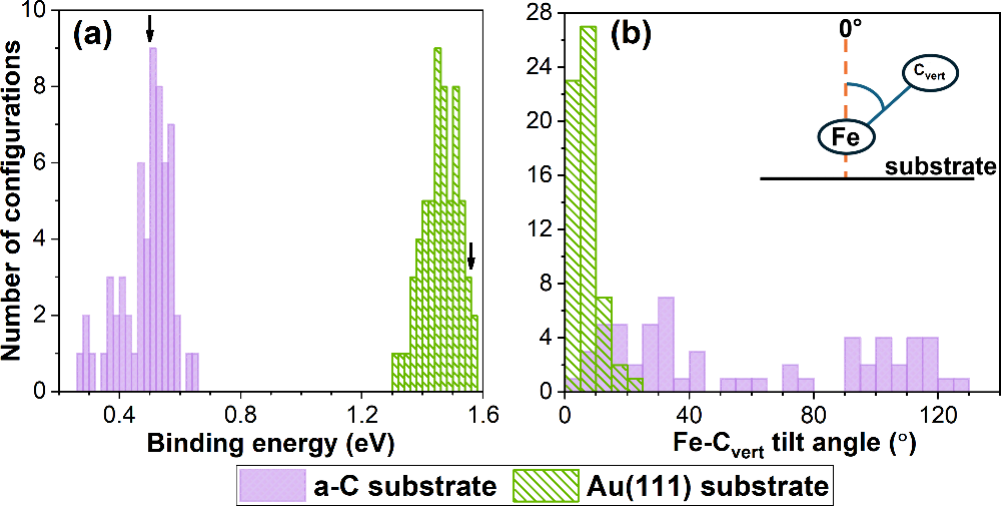
Structural characteristics and energetics of Fe­(CO)_5_^+^ adsorption on Au(111) and amorphous carbon (a-C) substrates. (a) The variation of the binding energies of Fe­(CO)_5_^+^ to each substrate. Shaded bars show the number of sampled configurations, while black arrows mark binding energies corresponding to the optimized geometries obtained using the classical interatomic potentials: 1.56 eV for Au(111) and 0.50 eV for a-C. The bin width is 0.02 eV. (b) The variation of the Fe–C_vert_ bond orientation relative to the normal to the substrate (see a schematic image in the inset). C_vert_ denotes the carbon atom in a nominally vertical CO ligand, which is almost orthogonal to the four other ligands (see Figure 1a). The tilt angle of 0° corresponds to a perfectly upright CO bond, perpendicular to the substrate surface. The bin width is 5°. The results shown in both panels are based on 60 independent initial configurations of each system obtained from MD trajectories.

Figure 2a shows the variation of the binding energies of the Fe­(CO)_5_^+^ cation on Au(111) and a-C substrates for different geometrical configurations. According to the performed calculations, the binding energy between Fe­(CO)_5_^+^ and the Au(111) substrate, corresponding to the optimized geometry, is ∼1.56 eV, indicating a relatively strong binding to the substrate. This suggests that the optimized configuration corresponds to a stable adsorption site on top of the ordered gold surface (see Figure 1b). The crystallographic orientation of gold surfaces affects the strength of molecular binding: the binding energy between Fe­(CO)_5_^+^ and the Au(100) surface is ∼1.36 eV (see Figure S4). Finite-temperature MD simulations yield adsorption sites that are predominantly less energetically favorable (with binding energies ranging from ∼1.3 to 1.58 eV) due to thermal vibrations of atoms in the molecule and the substrate. Overall, the binding energies of Fe­(CO)_5_^+^ and the three considered gold substrates are within the range of ∼1.0 – 1.6 eV, with the interaction with Au(111) being the strongest (see the Supporting Information, Figure S4). In contrast, the binding energy between Fe­(CO)_5_^+^ and the a-C substrate is notably weaker (see Figure 2a), and the optimized geometry corresponds to the binding energy of ∼0.50 eV. Finite-temperature MD simulations of Fe­(CO)_5_^+^ on a-C reveal both less and more energetically favorable adsorption sites (with binding energies ranging from ∼0.25 eV to ∼0.65 eV), which reflects the existence of many possible molecular orientations and configurations on the uneven carbon surface. A similar feature has also been observed in finite-temperature MD simulations of Fe­(CO)_5_^+^ on the polycrystalline Au substrate (see Figure S4).

Figure 2b shows the variation of the tilt angle between the axis connecting the Fe and C_vert_ atoms (see Figure 1a) and the normal to the substrate (aligned with the *z*-axis). On Au(111), as well as on the other considered gold substrates, the angular distribution is narrow and centered close to 0°, indicating a well-defined upright orientation of the “vertical” CO ligand containing the C_vert_ atom. In contrast, the angular distribution on the a-C substrate is much broader, reflecting the greater variation in Fe­(CO)_5_^+^ adsorption configurations on the disordered substrate. As discussed below, these structural differences directly impact the fragmentation behavior of the deposited molecule and the desorption of the parent molecule and produced fragments.

### Fragment Abundance Distribution

The results of the IDMD simulations of radiation-induced Fe­(CO)_5_^+^ fragmentation are summarized in Figure 3, which shows the relative abundances of Fe­(CO)_5–*n*_^+^ species (*n* = 0–5) as a function of the excess energy deposited into the parent cation. The results are presented for two substrates – Au(111) and amorphous carbon – and two energy transfer mechanisms described in the previous section. The corresponding results for the Au(100) and polycrystalline gold substrates are given in the Supporting Information (Figure S5). In the case of the localized energy transfer mechanism, we report the results for the energy deposition into the Fe–C_vert_ bond (panels (c,d)) and into one of Fe–C_hor_ bonds (panels (e,f)). For the sake of comparison, we also show two of our previously published fragmentation patterns:^18^ for an isolated Fe­(CO)_5_^+^ cation (thermal mechanism, Figure 3g) and for Fe­(CO)_5_^+^ embedded in an argon cluster of ∼250 atoms (localized energy deposition, Figure 3h).

**Figure 3:**
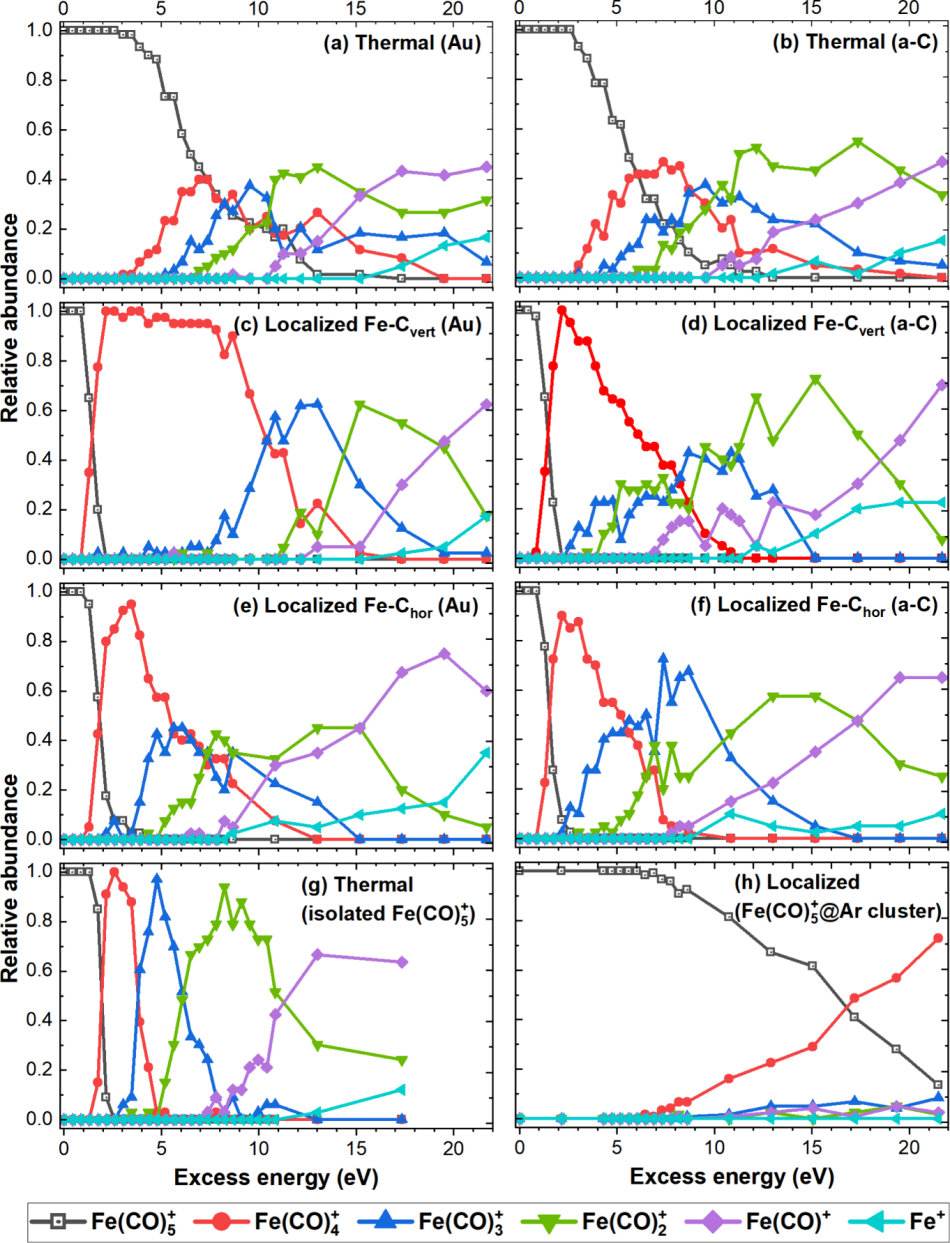
Relative abundances of the parent Fe­(CO)_5_^+^ ion and Fe­(CO)_*n*_^+^ (*n* = 0 – 4) fragments as a function of the energy transferred to the parent cation, calculated using IDMD. Panels (a–f) show the results obtained in this study. The left column (panels (a, c, e)) corresponds to a crystalline Au(111) substrate, while the right column (panels (b, d, f)) corresponds to an amorphous carbon (a-C) substrate. Panels (a, b) (top row) correspond to the thermal energy deposition mechanism; panels (c, d) (second row) to the localized energy transfer into the Fe–C_vert_ bond, and panels (e, f) (third row) to the localized energy deposition into one of the Fe–C_hor_ bonds. Panels (g, h) (the bottom row) show the abundances of molecular fragments^18^ due to the thermal energy deposition into an isolated Fe­(CO)_5_^+^ ion (panel (g)) and the localized energy deposition in Fe­(CO)_5_^+^ embedded into an argon cluster (panel (h)).

As expected, all abundance distributions have several general features. Very low excess energies (typically, up to 1–3 eV) are insufficient to dissociate the parent ion, and only intact Fe­(CO)_5_^+^ species are observed for different energy deposition mechanisms (black curves). As excess energy increases, more ligands are cleaved from the iron atom. However, the fragment abundance distributions differ significantly for different systems (an isolated molecule vs a molecule embedded in a cluster vs a molecule deposited on a surface) and different energy deposition scenarios.

Table 1 compares the values of the appearance energies (AEs) for Fe­(CO)_5–*n*_^+^ (*n* = 0–5) species obtained in this study and also in previous experimental and computational studies.^18,29^ The listed data corresponds to the present simulation results for the thermal and localized energy transfer mechanism (for both Fe–C_vert_ and Fe–C_hor_ cases) for the Au(111) and a-C substrates. These data are complemented with the results of our previous IDMD simulations^18^ for an isolated Fe­(CO)_5_^+^ cation (thermal energy transfer mechanism), DFT calculations performed in this study for isolated Fe­(CO)_5_^+^ (see the Supporting Information) and the AE values from gas-phase experiments by Lacko et al.^29^ It should be noted that the simulations were performed with an excess energy increment of ∼0.43 eV (10 kcal/mol), and the reported AE corresponds to the last energy point before the fragment abundance curve begins to rise (see Figure 3).

**Table 1:** Appearance Energies of Fragment Ions Obtained from the Present IDMD Simulations (The First Six Lines)^a^

	Fe(CO)_4_^+^	Fe(CO)_3_^+^	Fe(CO)_2_^+^	Fe(CO)^+^	Fe^+^
Thermal – Au	3.47	4.77	6.50	10.40	15.18
Thermal – a-C	2.60	3.90	5.64	9.54	17.34^a^
Localized (Fe–C_vert_) – Au	0.87	7.37^a^	10.84	12.14^a^	15.18^a^
Localized (Fe–C_vert_) – a-C	0.87	2.17	3.90	6.50	11.27^a^
Localized (Fe–C_hor_) – Au	0.87	3.04	4.77	7.81	8.24
Localized (Fe–C_hor_) – a-C	0.87	1.17	4.77	7.37	8.67
Thermal (isolated Fe(CO)_5_^+^)^18^	1.20	3.50	4.70	7.00	11.00
DFT (isolated Fe(CO)_5_^+^)	0.97	2.54	4.37	6.03	8.15
Exp. (gas phase)^29^	1.05	2.09	2.91	4.52	6.24

a For comparison, the following complementary data is presented: fragment appearance energies from earlier IDMD simulations^18^ of the fragmentation of isolated Fe­(CO)_5_^+^, reaction energies calculated in this study using DFT at the B3LYP/6-31+G­(d) level, and the appearance energies obtained from gas-phase experiments^29^ on electron-impact dissociative ionization of Fe­(CO)_5_. The energies are listed with respect to the ground state of the parent Fe­(CO)_5_^+^ cation. All values are given in eV.

b Indicates higher degree of uncertainty

A consistent trend in the simulations corresponding to the thermal energy transfer mechanism is that the presence of a substrate results in higher appearance energies than in simulations of an isolated molecule. This can be seen by comparing the fragment abundance profiles in Figures 3(a,b) and in Figure 3g. Interestingly, the fragmentation patterns are similar for both Au and a-C substrates. While there are well-defined energy thresholds for opening of each fragmentation channel, the relative abundance of different Fe­(CO)_5–*n*_^+^ fragments remains similar across a wide range of excess energies. Unlike the fragmentation of an isolated molecule^18^ (see Figure 3g), there are no characteristic excess energy ranges for the formation of a specific fragment. This suggests that, in the thermal energy transfer mechanism, different fragmentation pathways compete when the precursor molecule is adsorbed onto a surface. Complete loss of all CO ligands (i.e., formation of a Fe^+^ ion) remains rare, which is consistent with the earlier results for the gas phase.^18^

It is interesting to compare the results obtained in this study with earlier results^18^ describing the fragmentation of an Fe­(CO)_5_^+^ ion embedded in an argon cluster, see Figure 3h for the localized energy transfer mechanism. The shift in fragment appearance energies and quenching of Fe­(CO)_5_^+^ fragmentation in an argon environment are much more pronounced than for Fe­(CO)_5_^+^ physisorbed on the two substrates considered in this study. This indicates that the transfer of the excess energy from a Fe­(CO)_5_^+^ ion to the argon environment is much more effective. This could be due to the lower vibrational temperature of the argon cluster (in our previous simulations,^18^ the argon cluster was thermalized at 40 K before picking up the Fe­(CO)_5_^+^ ion, in accordance with the conditions of the corresponding experiments^38^) and the soft nature of argon vibrations (which correspond to a high density of vibrational states). Atomic and molecular clusters are often considered to be suitable proxies for macroscopic surfaces.^70,71^ However, the observed differences demonstrate that, while this is true in a qualitative sense, there may be significant quantitative differences.

Several additional factors must be considered for the localized energy transfer mechanism. According to the performed DFT calculations, the bond dissociation energy (BDE) of the Fe–C_vert_ bond in Fe­(CO)_5_^+^ is approximately 1.14 eV, whereas the BDE for Fe–C_hor_ bonds is around 1.56 eV. Due to the excess energy increment of 0.43 eV used in the IDMD simulations, the fragment appearance energies listed in Table 1 are lower than the actual BDE values.

As described in the Supporting Information, Fe­(CO)_4_^+^ can adopt two minimum-energy geometries depending on whether a Fe–C_vert_ or a Fe–C_hor_ bond in the parent ion is cleaved: (i) an almost planar structure resulting from loss of the “vertical” CO ligand containing the C_vert_ atom, and (ii) a “roof-like” geometry formed after losing a “horizontal” ligand containing a C_hor_ atom (see Figure S2). The orientation of the “vertical” CO ligand significantly influences the fragmentation pathway, as illustrated in Figure 2b. An angle of 0° corresponds to a perfectly upright CO bond relative to the normal to the substrate surface. In the case of Fe­(CO)_5_^+^ on the Au(111) substrate, the “vertical” CO ligand is generally aligned with the *z*-axis (see Figure 2b). In contrast, on the a-C substrate, the Fe–C_vert_ tilt angle varies significantly, often exceeding 90°, due to the strong rearrangement and rotation of Fe­(CO)_5_^+^ on the uneven a-C substrate (see Figure 1c) and the weaker binding of the Fe­(CO)_5_^+^ ion to the substrate.

Now, let us discuss the fragment abundance distribution for the case of Fe­(CO)_5_^+^ on the Au(111) substrate. In the localized mechanism of energy transfer into the Fe–C_vert_ bond, the appearance energy for the Fe­(CO)_3_^+^ ion is significantly higher than the appearance energy of Fe­(CO)_4_^+^ (see Figure 3c). The cleavage of the Fe–C_vert_ bond in the parent ion results in the formation of a stable planar Fe­(CO)_4_^+^ structure. The ordered Au(111) substrate prevents significant structural rearrangement of the Fe­(CO)_4_^+^ fragment, and its remaining excess energy is efficiently dissipated to the substrate. Consequently, no further fragmentation occurs for the excess energies up to 7 eV (Figure 3c). This fragmentation pathway is a distinctive feature arising from the relatively strong nonbonded interaction between the fragment ion and the ordered crystalline gold substrate.

In contrast, the structural irregularity of the a-C substrate prevents the formation of a stable planar Fe­(CO)_4_^+^ structure. This results in lower AEs for Fe­(CO)_4_^+^ and smaller fragments (Figure 3d), as well as in a broader distribution of the fragment abundances over a wider range of excess energies, compared to the localized energy transfer mechanism on the Au(111) substrate (Figure 3c) and the thermal mechanism on both substrates (Figures 3a,b). A similar trend is observed for the localized energy transfer into one of the Fe–C_hor_ bonds (Figure 3f): the fragment appearance energies shift to lower values, and the dominant fragmentation pathway involves the loss of a single CO ligand at energies close to the corresponding BDE.

### Impact of Charge-Induced Polarization Effects on Fragmentation Dynamics

The results presented in the previous sections were obtained without explicitly accounting for polarization effects induced by a charged molecular species located in proximity to a conductive substrate (particularly gold). Inclusion of this additional attractive interaction between the molecular ion and the substrate may be important for a more accurate quantitative description of adsorption energetics and surface dynamics. In principle, such effects can be treated using more advanced quantum chemistry calculations accounting for long-range dispersion corrections, as well as dedicated polarizable force fields;^72–74^ however, this approach lies beyond the scope of the present work.

To mimic the attractive interaction of a molecular ion with a metallic substrate, we introduced an effective atom–surface polarization term of the form – C_4_/*r* ^4^ (see details in the Supporting Information). Using the atomic partial charges for Fe­(CO)_5_^+^, we evaluated the coefficients of the effective image-type polarization potential by matching its continuum limit to the exact image interaction above a metallic plane: *C*_4_ (Fe–Au) = 1.26 eV Å^4^, *C*_4_ (C–Au) = 3.31 eV Å^4^, and *C*_4_ (O–Au) = 0.49 eV Å^4^. The obtained parameters reproduce the self-image contribution of each atomic charge center in the long-range limit and provide a computationally efficient approximation to metallic polarization within a nonpolarizable force-field framework.

Figure 4a illustrates the effect of including the polarization interaction on the binding energy of Fe­(CO)_5_^+^ on the Au(111) substrate. The additional attractive interaction increases the binding energy of the molecular ion by ∼1 eV. As discussed in the following subsection, this enhanced stabilization results in an approximately 2-fold decrease in the desorption probability of Fe-containing fragments from the Au(111) substrate.

**Figure 4:**
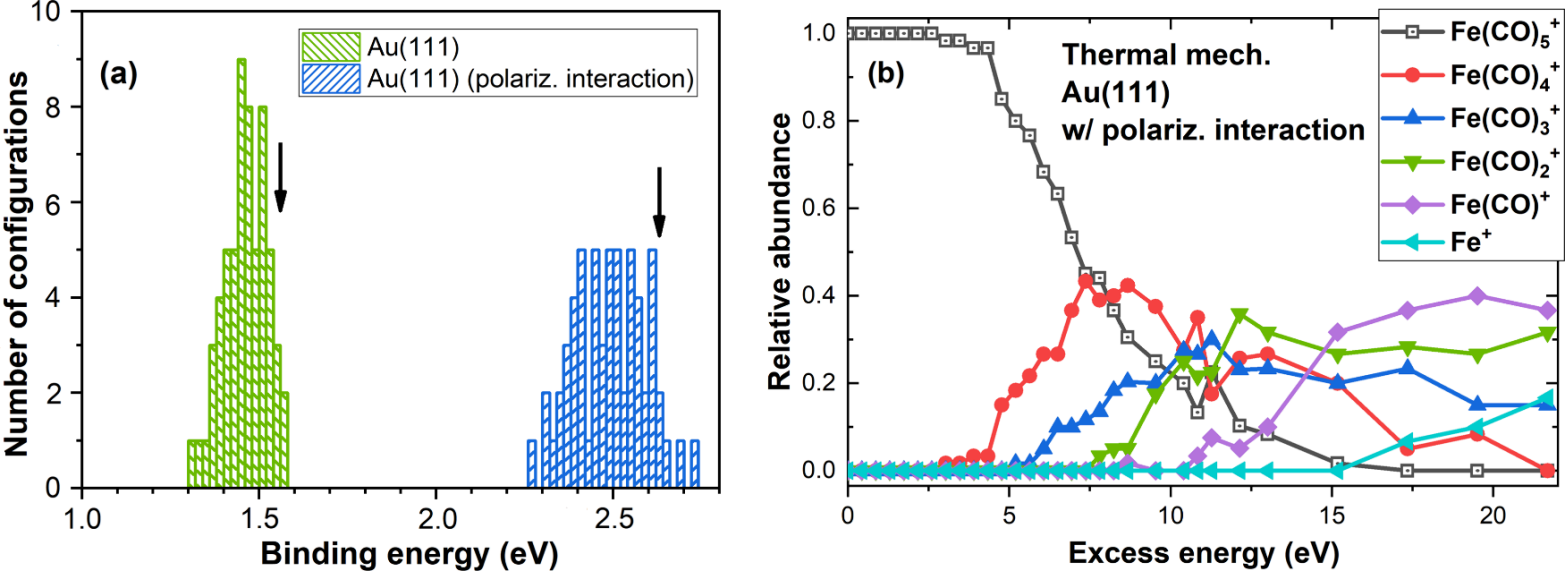
Results of the simulations including the attractive polarization interaction between the Fe­(CO)_5_^+^ ion and the Au(111) substrate. (a) The variation of the binding energy. Shaded bars show the number of sampled configurations, while black arrows mark binding energies corresponding to the optimized geometries obtained using the classical interatomic potentials. (b) Relative abundances of the parent Fe­(CO)_5_^+^ ion and Fe­(CO)_*n*_^+^ (*n* = 0–4) fragments as a function of the energy transferred to the parent cation, calculated using IDMD.

Despite this significant increase in binding energy, the polarization interaction has a minor effect on the overall fragment abundance distribution. Figure 4b shows the distribution of fragment abundances obtained for the thermal mechanism of energy deposition on the Au(111) substrate with the polarization interaction included. Comparison with the corresponding distribution shown in Figure 3a reveals a high degree of similarity between the two cases. In the new simulations, the appearance energy of Fe­(CO)_4_^+^ fragments increases by ∼1 eV, corresponding to an increase in the binding energy of Fe­(CO)_5_^+^ to the substrate. Apart from that, only a slight reduction (less than 10%) is observed in the abundances of intermediate and small fragments, such as Fe­(CO)_3_^+^, Fe­(CO)_2_^+^, and Fe­(CO)^+^. These results suggest that the energy deposited into the parent ion is sufficient to induce desorption irrespective of the additional polarization interaction, and that the initial fragmentation events predominantly occur above the substrate. At the same time, the reduced abundance of smaller fragments indicates that the attractive polarization interaction promotes readsorption of these species onto the surface, thereby suppressing subsequent fragmentation pathways that would otherwise occur in the gas phase.

### Probability of Fragment Desorption from the Substrate

Figure 5 shows how the internal energy of the deposited Fe­(CO)_5_^+^ molecule is dissipated over time into the Au(111) and a-C substrates for different energy deposition mechanisms. The *y*-axis represents the normalized kinetic energy (KE) of all the atoms in the parent Fe­(CO)_5_^+^ ion or in the resulting fragments if the parent ion has fragmented. To analyze the redistribution of the excess energy deposited in the molecule by irradiation, the value of ∼0.4 eV, corresponding to the KE of a Fe­(CO)_5_^+^ ion at 300 K, was subtracted. Each curve in Figure 5 represents the average of 60 independent configurations obtained from the MD simulations. The range of excess energies considered, from ∼0.87 eV (20 kcal/mol) to ∼3.90 eV (90 kcal/mol), has been chosen to focus on the intact parent ion and fragments with a low degree of fragmentation. The curves plotted in Figure 5 therefore show the fraction of excess energy remaining in the deposited species over time, and how efficiently this energy is transferred to the Au(111) and a-C substrates. The general trend is that the physisorbed cation rapidly loses most of the excess energy within the first ∼20 ps of the simulations. This dissipation occurs slightly faster on the Au substrate than on the a-C substrate, likely due to more effective energy transfer to the crystalline lattice. In all cases, the remaining kinetic energy after 200 ps of the simulation stabilizes at approximately 10–20% of the initially deposited energy. For the Au substrate, the rate of energy flow also depends on the initial energy deposition scenario. The rate is fastest for the statistical distribution (thermal mechanism), intermediate for the case where the energy is initially deposited in a bond parallel to the surface (Fe–C_hor_), and slowest if the energy is initially deposited in the bond pointing away from the surface (Fe–C_vert_). This result is in accordance with the intuitive expectation of the coupling of a particular motion to surface atoms.

**Figure 5:**
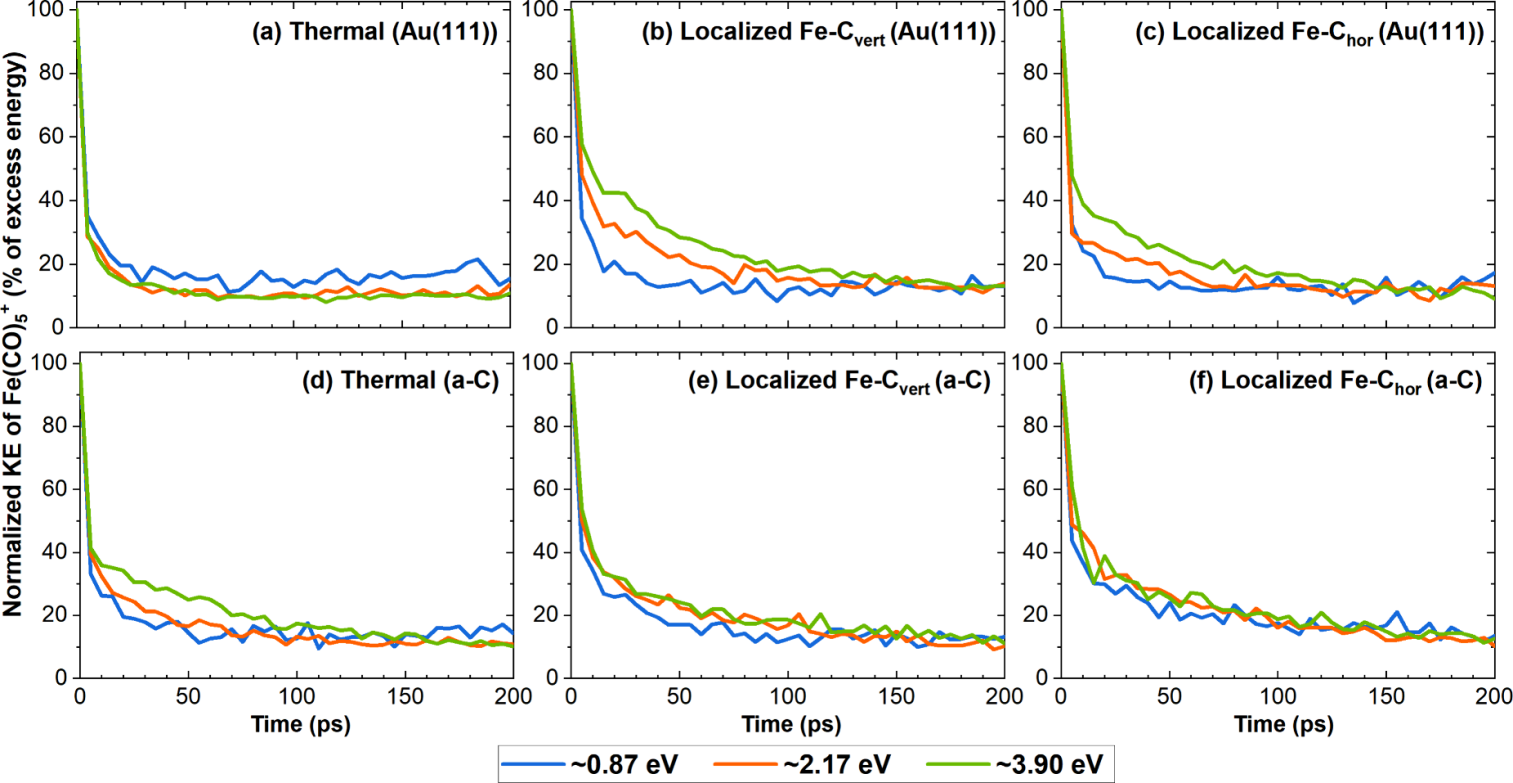
Time evolution of the kinetic energy (KE) of Fe­(CO)_5_^+^ expressed as a percentage of the excess energy initially deposited in the Fe­(CO)_5_^+^ parent cation by irradiation. The KE is calculated as the sum of the kinetic energies of all of the atoms in the intact cation or in all its molecular fragments if the parent ion has fragmented. A value of 100% corresponds to the full amount of excess energy deposited to the system, ranging from ∼0.87 eV (20 kcal/mol) to ∼3.90 eV (90 kcal/mol). The decay reflects how much of that energy remains in the deposited molecule over time. To normalize the amount of dissipated energy, the kinetic energy of the Fe­(CO)_5_^+^ parent cation at 300 K (approximately 0.4 eV) was subtracted from all KE values. The resulting values were then expressed as a percentage of the excess energy for each case. Panels (a–c) show the results for the Au(111) substrate; panels (d–f) describe the case of the amorphous carbon (a-C) substrate.

The energy flow to the substrate also affects the desorption of fragments. This is illustrated in Figure 6, which shows the relative probability of desorption of Fe-containing species, Fe­(CO)_5–*n*_^+^ (*n* = 0 – 5), as a function of excess energy. This function is defined as the ratio of the number of desorbed Fe-containing species to the total number of performed IDMD simulations. Figure 6a shows the results for the thermal energy deposition mechanism. On the a-C substrate (purple symbols), the desorption of fragments occurs at excess energies of ∼2 eV, and the desorption probability increases sharply above this value. In contrast, desorption of fragments from the Au(111) substrate (green symbols) occurs with a significantly lower probability across the entire range of excess energies considered. Accounting for the polarization interaction induced by the molecular ion in the Au(111) substrate results in an approximately 2-fold decrease of the fragment desorption probability (see blue symbols in Figure 6a). As discussed above, a possible explanation for this effect is that the desorbed fragments do not leave the surface completely but readsorb due to the additional attractive interaction due to the polarization effects.

**Figure 6:**
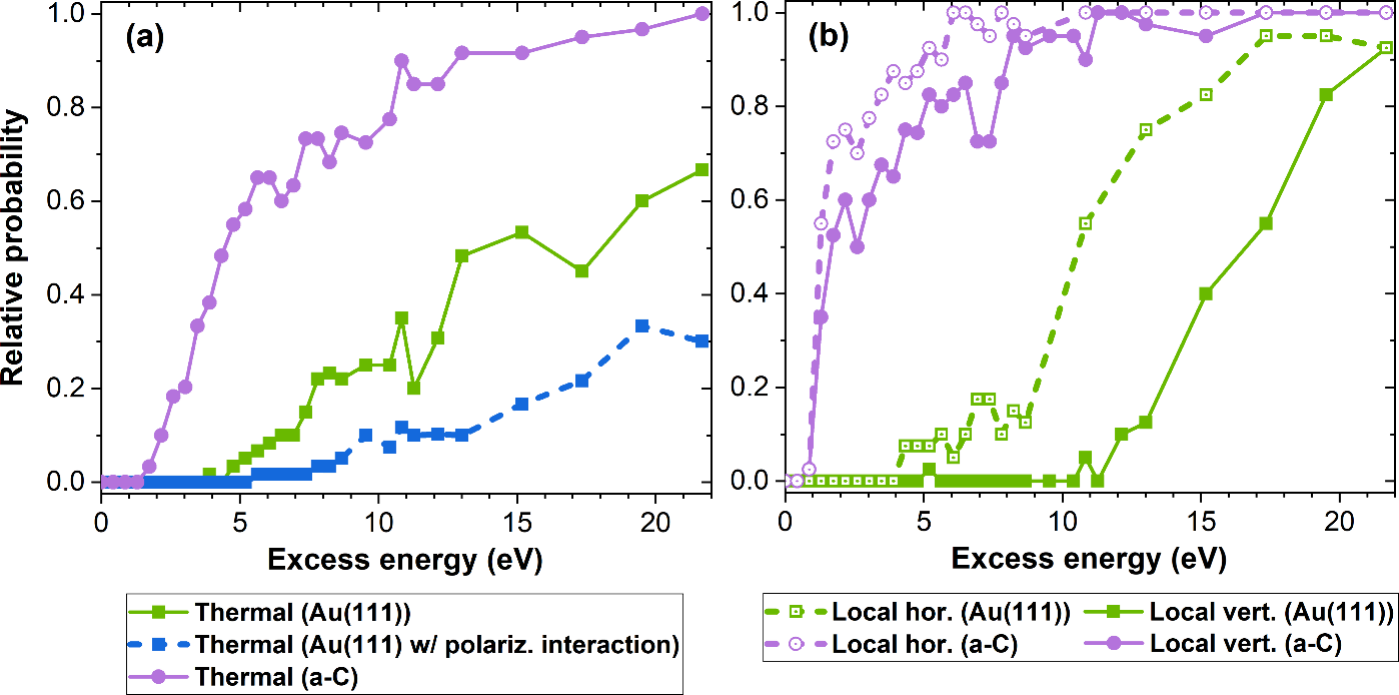
Relative probability of the detachment of Fe-containing species, Fe­(CO)_5–*n*_^+^ (*n* = 0–5), from the Au(111) and a-C substrates as a function of excess energy. The plotted probability is defined as the ratio of the number of desorbed Fe-containing species to the total number of performed IDMD simulations. Panel (a) shows results for the thermal energy transfer mechanism, while panel (b) shows results for the localized energy transfer mechanism. The notations “Local hor.” and “Local vert.” refer to the localized energy deposition into a Fe–C_hor_ and the Fe–C_vert_ bonds, respectively.

It should be noted that the simulations of Fe­(CO)_5_^+^ dynamics and desorption on the a-C substrate have been performed on an unpassivated carbon surface. In principle, such a substrate contains reactive sites capable of forming covalent bonds with Fe-containing fragments, leading to chemisorption. In the present work, however, we have focused on physisorption processes and therefore have not allowed for the formation of covalent bonds between the fragments and substrate atoms. Despite this approximation, the results obtained for the a-C substrate (Figure 6) remain quantitatively reliable. Analysis of the simulated IDMD trajectories reveals three main dynamical pathways for Fe­(CO)_5_^+^. In the first scenario, the intact molecule diffuses along the substrate and remains adsorbed throughout the simulation. In the second scenario, the Fe­(CO)_5_^+^ molecule desorbs from the surface and subsequently fragments in the gas phase at distance of several angstroms from the substrate. The resulting fragments, including Fe-containing species, then propagate freely within the simulation box without readsorbing on the time scale considered. In the third scenario, fragmentation occurs (in most cases, above the surface), followed by rapid readsorption of the fragments, which then remain bound to the substrate. Explicit inclusion of the probability of chemisorption would likely reduce the probability of repeated desorption events in the latter case by stabilizing Fe-containing fragments on the surface. However, analysis of the trajectories indicates that such events are relatively infrequent and therefore do not significantly affect the overall results shown in Figure 6.

Figure 6b shows the desorption probabilities of Fe-containing fragments for the localized mechanism of energy transfer into the Fe–C_vert_ bond and one of the Fe–C_hor_ bonds. On the Au(111) surface, both scenarios result in a low (<20%) fragment desorption probability for excess energies up to ∼10 eV and even up to ∼13 eV for energy deposition into the Fe–C_vert_ bond. In contrast, on the a-C substrate, desorption of Fe-containing fragments begins at much lower excess energies (around 1–2 eV) and the desorption probability increases to (80–100)% at excess energies above 5 eV. Interestingly, the localized energy deposition into a Fe–C_hor_ bond results in a higher fragment desorption probability than the energy deposition into the Fe–C_vert_ bond, and this has been observed for both substrates. This suggests that molecular fragments formed by cleavage of a Fe–C_hor_ bond are less stable (see Figure S2 in the SI) and can desorb more easily.

It should be noted that Figure 6 shows the cumulative desorption probability over all Fe-containing fragments. This is motivated by the recent study,^21^ discussed below, which addressed the desorption of charged, metal-containing fragments from the substrate. In the SI, we present the current desorption results, broken down for each particular fragment (Figure S7).

## Conclusions

In conclusion, we have presented the results of irradiation-driven molecular dynamics simulations of the fragmentation of iron pentacarbonyl cation physisorbed on several different substrates – oriented Au(111) and Au(100), polycrystalline gold, and amorphous carbon. These results demonstrate that the geometrical characteristics and irregularities of the substrate as well as the strength of molecular binding to the substrate determine the efficiency of energy transfer and the subsequent release of metal-containing fragments. The amorphous carbon substrate, with its higher degree of structural disorder and weaker interaction with the deposited Fe­(CO)_5_^+^ molecule, facilitates the desorption of Fe-containing molecular fragments. In contrast, the ordered and stronger-interacting Au(111) and Au(100) substrates stabilize the adsorbed molecule and suppress the desorption of charged Fe-containing fragments, particularly in the localized mechanism of energy transfer to the deposited molecule. This substrate-dependent behavior significantly alters fragmentation pathways and fragment desorption probabilities, providing valuable insights into the interactions between the Fe­(CO)_5_ precursor molecule and experimentally relevant surfaces.

The results presented in this study have several implications. First, we have demonstrated the importance of explicit accounting for the energy transfer from deposited precursors to the substrate. These results can be used to further advance atomistic simulations of the FEBID process with IDMD.^11–15^ Previous IDMD simulations of FEBID have not explicitly considered the energy transfer between the excited precursor molecules and the substrate. The corresponding quenching of the fragmentation, which we have put here on a quantitative basis, may affect the chemical composition and metal content of the simulated deposits. The second implication concerns the desorption of ionic species from the substrate. This process is central to the microanalytical focused-electron-beam-induced mass spectrometry (FEBiMS) technique,^75^ which relies on *in situ* monitoring of charged fragments released from the irradiated substrate under typical FEBID conditions (a constant supply of precursor via a gas injection system; a focused 10 keV electron beam; and no postionization of the released material). A recent experimental and computational study^21^ has presented evidence of a high degree of similarity between FEBiMS and gas-phase fragmentation mass spectra, and has also reported high binding energies of metal-containing cations to a SiO_2_ substrate, as determined by DFT calculations. As the binding energies of cationic species are much larger than those of the corresponding neutral fragments, these findings suggested, based on an Arrhenius-type dependence, that the desorption of *charged* species from the surface is highly improbable.^21^ Through the IDMD simulations performed in this study, we demonstrate that binding energy is only one factor governing the desorption of molecular ions. Another important factor is the redistribution of excess energy (deposited into the physisorbed molecule by irradiation) to the substrate, which strongly enhances the probability of fragment desorption.

## Supplementary Material



## Notes

Details of the DFT calculations and benchmarks, rCHARMM force field parameters, details about creation of gold and amorphous carbon substrates, description of the polarization effects, comparison of the dynamics on Au(111), Au(100), and polycrystalline Au substrates, and description of fragment-resolved desorption probabilities (PDF)
