# Supplementary material for: Atomistic Simulations of Fe(CO)5 Fragmentation Dynamics on a Substrate
Source: J Phys Chem C Nanomater Interfaces. 2026 Jun 4;130(26):9102–13. doi: 10.1021/acs.jpcc.5c08601 (PMC13339783; doi:10.1021/acs.jpcc.5c08601)
Supplement: Supplementary file 1 [file jp5c08601_si_001.pdf]

Supporting Information for Atomistic Simulations of $Fe(CO)_5$ Fragmentation Dynamics on a Substrate

Hlib Lyshchuk,[†] Alexey V. Verkhovtsev,[*,¶] Juraj Fedor,[*,†] and Andrey V. Solov'yov[¶]

†*J. Heyrovský Institute of Physical Chemistry, Czech Academy of Sciences, Dolejškova 3, 18223 Prague, Czech Republic*

‡*Department of Physical Chemistry, University of Chemistry and Technology, Technická 5, 16628 Prague, Czech Republic*

¶*MBN Research Center, Altenhöferallee 3, 60438 Frankfurt am Main, Germany*

E-mail: verkhovtsev@mbnresearch.com; juraj.fedor@jh-inst.cas.cz

DFT calculations and benchmarks

Density functional theory (DFT) calculations were performed using the Gaussian 16 software package[1] with the hybrid B3LYP functional,[2] the 6-31+G(d) basis set,[3,4] and the GD3 empirical dispersion correction.[5] The calculations were performed to determine the parameters of the reactive rCHARMM force field[6] (see the next section) for IDMD simulations of $Fe(CO)_5^+$ fragmentation. Geometry optimization calculations were carried out for both the neutral and cationic parent species, $Fe(CO)_5$ and $Fe(CO)_5^+$, as well as for the molecular fragments $Fe(CO)_n^+$ ($n = 0 - 4$). The obtained optimized geometries were then used to identify the most probable reaction channels by evaluating the appearance energies (AEs) of the fragments associated with each fragmentation pathway.

From the optimized structures, relaxed potential energy scans were performed for different covalent bonds (Fe–C_{vert}, Fe–C_{hor}, C_{vert}–O and C_{hor}–O; see Figure 1a in the main text) and angles in $Fe(CO)_n^+$ ($n = 0-4$) species. For each reaction coordinate, the corresponding bond length or angle was incrementally varied by 0.1 Å or 1°, respectively. Geometry optimization was then performed at each point. The resulting potential energy curves were fitted with the Morse potential, see Eq. (S1) below, to determine bond dissociation energies (BDEs) and force constants. The determined rCHARMM parameters for the bonded and angular interactions are summarized in Tables S3 and S4.

Additionally, Natural Bond Orbital (NBO) analyses were performed to obtain information on the atomic partial charges in the parent cation and its fragments. These calculations were performed to dynamically adjust the interaction parameters during the IDMD simulations of $Fe(CO)_5^+$ fragmentation. The ability to change atom types and their interaction parameters is a distinctive feature of the IDMD[7] and rCHARMM[6] methods implemented in MBN Explorer.[8] This approach, illustrated in our previous study,[9] enables an accurate capture of the molecular response to irradiation and, consequently, a realistic description of molecular fragmentation pathways. The resulting NBO-derived partial charges are listed in Table S5.

To verify the utilized computational approach and confirm the validity of the rCHARMM parametrization for $Fe(CO)_5^+$, geometry optimization calculations of the precursor were also performed on two nano-supports relevant to this study: a 1-atom-thick Au(111) patch containing 25 atoms and an ovalene ($C_{32}H_{14}$) molecule as a model for the carbon substrate (see Figure S1). In both cases, the substrate atoms were fixed at their equilibrium positions during optimization to reduce computational cost. Higher-level calculations at the MP2/aug-cc-pVTZ and M06-2X/Def2TZVP levels of theory were also performed for the isolated $Fe(CO)_5^+$ molecule to provide a more rigorous comparison. These methods are too computationally demanding to be used for calculations involving nano-supports. The key geometrical parameters obtained from these calculations, such as the bond lengths and angles, are summarized in Tables S1 and S2 and are compared with the optimized geometries calculated using the rCHARMM force field.

For both adsorbed systems, the bond lengths obtained with different basis sets are consistent with each other (with relative differences below 0.03 Å) and agree also with geometries obtained from classical optimization calculations using MBN Explorer (with deviations below 0.09 Å for Fe–C bonds and 0.03 Å for C–O bonds). In the calculations performed for an isolated $Fe(CO)_5^+$ molecule, the Fe–C_{vert} bond length varies by approximately 0.4 Å depending on the method and basis set, ranging from 1.762 Å at the MP2/aug-cc-pVTZ level to 2.155 Å with M06-2X/Def2TZVP. Given the minimal variation between the results of different DFT approaches in the adsorbed case, the B3LYP/6-31+G(d) parametrization provides a consistent and adequate description of the molecular geometry of $Fe(CO)_5^+$ in the considered substrate environment.

The equilibrium angles in $Fe(CO)_5^+$, obtained using different basis sets and methods, are consistent for the isolated molecule and for the molecule placed on top of ovalene and Au(111), with deviations below 3°. A somewhat larger discrepancy is observed between the geometries obtained from classical and DFT optimizations. For $Fe(CO)_5^+$ on Au(111), the C_{hor1}–Fe–C_{hor1} angles differ by approximately 10°, which can be attributed to the difference

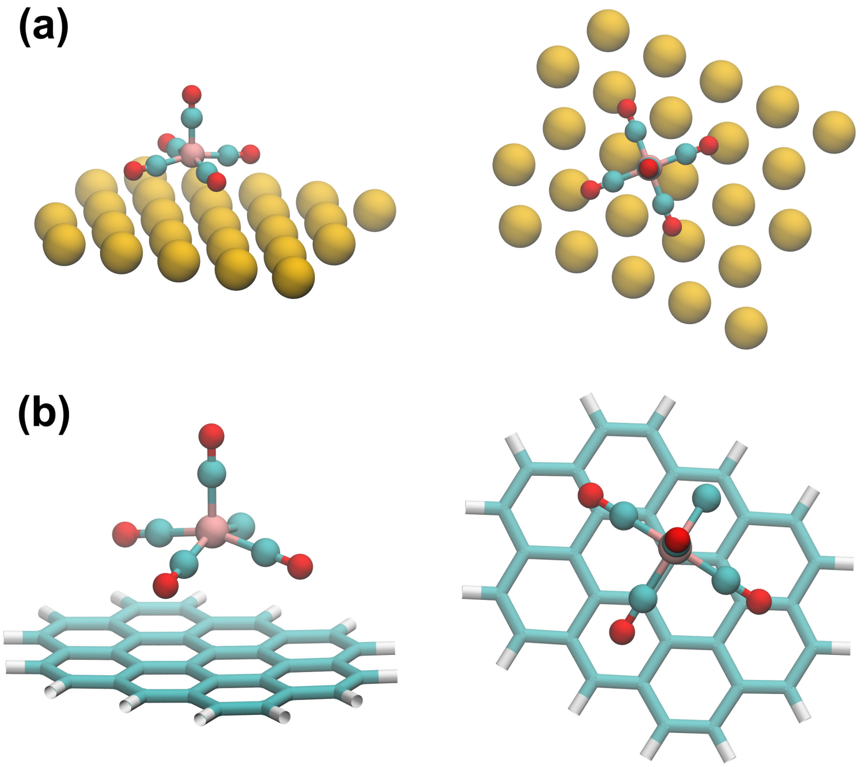

Figure S1: The optimized geometry of $Fe(CO)_5^+$ on a 25-atom Au(111) nano-support (panel (a)) and on an ovalene ($C_{32}H_{14}$) molecule (panel (b)), calculated using DFT at B3LYP/LANL2DZ level of theory. The left and right columns show three-dimensional projections and top views of each system, respectively.

in substrate representation. The classical optimization calculations performed using MBN Explorer employed a three-layer gold slab (see Figure 1b in the main text), whereas the DFT calculations used the frozen top layer, resulting in a different local environment for the adsorbed precursor. The difference is larger for the ovalene substrate (up to $\sim 17-20^\circ$) because the DFT calculations were performed for an ideal planar ovalene molecule as the substrate, whereas the MBN Explorer simulations were carried out on an amorphous carbon surface with an irregular, non-planar geometry (see Figure 1c in the main text).

Overall, the variations in the considered geometrical parameters across different DFT and MP2 calculations are comparable to the differences between the results of DFT and classical optimization calculations. This consistency confirms the suitability of the chosen DFT method for deriving the force-field parameters used in the classical simulations.

Table S1: The equilibrium bond lengths (in Å) for an isolated $Fe(CO)_5^+$ molecule and for $Fe(CO)_5^+$ adsorbed on the Au(111) and ovalene nano-supports, calculated using various DFT and post-HF methods. The atomic notations refer to the geometry of $Fe(CO)_5^+$, shown in Fig. S2.

Method / Basis set	Fe–C_{vert}	Fe–C_{hor1}	Fe–C_{hor2}	C_{vert}–O	C_{hor1}–O	C_{hor2}–O
**Free $Fe(CO)_5^+$:**						
B3LYP/6-31+G(d)†	1.944	1.898	1.898	1.135	1.135	1.135
MP2/aug-cc-pVTZ	1.762	1.764	1.764	1.143	1.146	1.143
M062X/Def2TZVP	2.155	2.001	2.001	1.114	1.114	1.114
B3LYP/LANL2DZ	1.939	1.882	1.882	1.157	1.157	1.157
**$Fe(CO)_5^+$ on Au(111):**						
B3LYP/LANL2DZ	1.805	1.815	1.816	1.168	1.171	1.171
B3LYP/Def2SVP	1.822	1.824	1.825	1.139	1.143	1.143
classical‡	1.896	1.880	1.883	1.133	1.134	1.133
**$Fe(CO)_5^+$ on Ovalene:**						
B3LYP/LANL2DZ	1.811	1.805	1.809	1.169	1.174	1.173
B3LYP/6-31+G(d)	1.833	1.815	1.820	1.146	1.152	1.150
B3LYP/Def2SVP	1.826	1.813	1.817	1.140	1.145	1.144
classical§	1.897	1.883	1.881	1.134	1.134	1.134

†Method and basis set used for parametrization in the present work

‡Results of the classical geometry optimization calculation on the Au(111) substrate

§Results of the classical geometry optimization calculation on the a-C substrate

Table S2: The equilibrium angles (in degrees) for an isolated $Fe(CO)_5^+$ molecule and for $Fe(CO)_5^+$ adsorbed on the Au(111) and ovalene nano-supports, calculated using various DFT and post-HF methods. The atomic notations refer to the geometry of $Fe(CO)_5^+$, shown in Fig. S2.

Method / Basis set	Fe–C_{vert}–O	Fe–C_{hor1}–O	Fe–C_{hor2}–O	C_{hor1}–Fe–C_{hor1}	C_{hor2}–Fe–C_{hor2}	C_{hor1}–Fe–C_{hor2}	C_{vert}–Fe–C_{hor1}	C_{vert}–Fe–C_{hor2}
**$Fe(CO)_5^+$ (gas phase):**								
B3LYP/6-31+G(d)†	180.0	180.0	180.0	167.3	167.3	89.3	96.4	96.4
MP2/aug-cc-pVTZ	180.0	178.9	178.9	167.9	167.9	89.4	96.0	96.1
M062X/Def2TZVP	180.0	179.6	179.6	169.8	169.8	89.6	95.1	95.1
B3LYP/LANL2DZ	180.0	180.0	180.0	167.7	167.7	89.3	96.1	96.1
**$Fe(CO)_5^+$ on Au(111):**								
B3LYP/LANL2DZ	179.9	176.1	175.9	162.7	162.1	88.7	98.7	99.0
B3LYP/Def2SVP	179.9	175.6	175.6	161.8	161.9	88.6	99.1	99.1
classical‡	180.0	177.9	179.0	172.9	174.5	89.3	93.6	92.8
**$Fe(CO)_5^+$ on Ovalene:**								
B3LYP/LANL2DZ	180.0	180.0	179.1	151.3	157.2	87.1	104.4	101.4
B3LYP/6-31+G(d)	180.0	179.7	178.5	149.1	157.7	87.0	105.4	101.1
B3LYP/Def2SVP	180.0	179.7	179.8	152.0	156.2	87.2	104.2	101.9
classical§	179.9	177.8	179.1	172.1	174.0	89.7	94.0	93.1

†Method and basis set used for parametrization in the present work

‡Results of the classical geometry optimization calculation on the Au(111) substrate

§Results of the classical geometry optimization calculation on the a-C substrate

rCHARMM force field parameters

In the performed irradiation-driven molecular dynamics (IDMD)[7] simulations of $Fe(CO)_5^+$ fragmentation, the interatomic interactions for $Fe(CO)_5^+$ and its fragments were described using the reactive rCHARMM force field introduced in Ref. 6. rCHARMM permits simulations of various molecular systems with the dynamically changing molecular topology,[7,9–14] which is essential for modeling irradiation-driven transformations and chemistry.

The radial part of the bonded interactions is described in rCHARMM by means of the Morse potential:

$$U^{\mathrm{bond}}(r_{ij}) = D_{ij}\left[e^{-2\beta_{ij}(r_{ij}-r_0)} - 2e^{-\beta_{ij}(r_{ij}-r_0)}\right]. \tag{S1}$$

Here D_{ij} is the dissociation energy of the bond between atoms i and j, r_0 is the equilibrium bond length, and the parameter $\beta_{ij} = \sqrt{k^r_{ij}/D_{ij}}$ (with k^r_{ij} being the bond force constant) determines the steepness of the potential. The bonded interactions are truncated at a user-defined cutoff distance beyond which the covalent bond gets broken and the molecular topology of the system changes.

The rupture of covalent bonds in the course of simulation employs the following reactive potential for valence angles:[6]

$$U^{\mathrm{angle}}(\theta_{ijk}) = 2k^{\theta}_{ijk}\,\sigma(r_{ij})\,\sigma(r_{jk})\left[1-\cos(\theta_{ijk}-\theta_0)\right], \tag{S2}$$

where θ_0 is the equilibrium angle formed by a triplet of atoms i, j and k; k^{θ} is the angle force constant; and the function

$$\sigma(r_{ij}) = \frac{1}{2}\left[1-\tanh(\beta_{ij}(r_{ij}-r^*_{ij}))\right] \tag{S3}$$

describes the effect of bond breakage, see Ref. 6 for the details. The parameter r^*_{ij} in Eq. (S3) is given by

$$r^*_{ij} = \frac{1}{2}\left(R^{\mathrm{vdW}}_{ij} + r_0\right), \tag{S4}$$

Table S3: Parameters of the covalent bonded interaction, Eq. (S1), for the $Fe(CO)_5^+$ parent ion and $Fe(CO)_n^+$ ($n = 1-4$) fragments. The atomic notations refer to the geometries of $Fe(CO)_5^+$, $Fe(CO)_4^+$ and $Fe(CO)_3^+$, shown in Fig. S2.

bond type	r_0 (Å)	k_{ij}^r (kcal/mol Å^{-2})	D_{ij} (kcal/mol)	D_{ij} (eV)
		**$Fe(CO)_5^+$:**		
Fe – C_{vert}	1.944	85.7	26.4	1.14
Fe – $C_{hor1,2}$	1.898	112.8	36.0	1.56
C_{vert} – O	1.135	1548.3	203.3	8.82
$C_{hor1,2}$ – O	1.135	1548.3	220.0	9.54
		**$Fe(CO)_4^+$ (non-planar):**		
Fe – C_{ax}	1.902	116.1	28.6	1.24
Fe – C_{hor}	1.904	116.1	36.0	1.56
C_{ax} – O	1.135	1548.3	256.1	11.10
C_{hor} – O	1.135	1548.3	256.1	11.10
		**$Fe(CO)_4^+$ (planar):**		
Fe – $C_{hor1,2}$	1.902	120.7	37.5	1.63
$C_{hor1,2}$ – O	1.135	1548.3	220.0	9.54
		**$Fe(CO)_3^+$:**		
Fe – C_{vert}	1.836	105.0	28.5	1.24
Fe – C_{hor}	1.920	105.0	35.5	1.54
C_{vert} – O	1.135	1548.3	256.1	11.10
C_{hor} – O	1.135	1548.3	256.1	11.10
		**$Fe(CO)_2^+$:**		
Fe – C	1.931	100.0	38.9	1.67
C – O	1.133	1548.3	256.1	11.10
		**$Fe(CO)^+$:**		
Fe – C	1.845	145.5	46.1	2.00
C – O	1.133	1548.3	256.1	11.10

where r_0 is the equilibrium distance between two atoms involved in the angular interaction and $R_{ij}^{\rm vdW}$ is the sum of the van der Waals radii for those atoms.

Table S3 lists the parameters for the bonded interactions, Eq. (S1). It includes information on the equilibrium bond lengths r_0, force constants k_{ij}^r, and dissociation energies D_{ij} for different bonds of the parent $Fe(CO)_5^+$ ion and the $Fe(CO)_n^+$ ($n = 1-4$) fragments. Table S4 lists the parameters for the angular interactions, Eq. (S2), for the $Fe(CO)_5^+$ parent ion and the $Fe(CO)_n^+$ ($n = 1-4$) fragments. It includes information on the equilibrium angles θ_0 and force constants k_{ijk}^{θ}. The corresponding atomic notations are shown in Fig. S2.

Table S4: Parameters of angular interactions, Eq. (S2), for the $Fe(CO)_5^+$ parent ion and the $Fe(CO)_n^+$ ($n = 1 - 4$) fragments. The atomic notations refer to the geometries of $Fe(CO)_5^+$, $Fe(CO)_4^+$ and $Fe(CO)_3^+$, shown in Fig. S2.

angle type	θ_0 (deg.)	k^{θ}_{ijk} (kcal/mol rad^{-2})
**$Fe(CO)_5^+$:**		
Fe–C_{vert}–O	180.0	28.0
Fe–$C_{hor1,2}$–O	180.0	28.0
C_{vert}–Fe–$C_{hor1,2}$	96.4	35.4
C_{hor1}–Fe–C_{hor2}	89.3	62.6
C_{hor2}–Fe–C_{hor2}	167.3	53.5
C_{hor1}–Fe–$_{hor1}$	167.3	53.5
**$Fe(CO)_4^+$ (non-planar) :**		
Fe–C_{ax}–O	176.8	28.0
Fe–C_{hor}–O	179.5	28.0
C_{ax}–Fe–C_{hor}	92.2	100.0
C_{hor}–Fe–C_{hor}	172.3	100.0
C_{ax}–Fe–C_{ax}	110.4	100.0
**$Fe(CO)_4^+$ (planar) :**		
Fe–$C_{hor1,2}$–O	180.0	28.0
C_{hor1}–Fe–C_{hor2}	90.0	100.0
C_{hor2}–Fe–C_{hor2}	180.0	100.0
C_{hor1}–Fe–$_{hor1}$	180.0	100.0
**$Fe(CO)_3^+$:**		
Fe–C_{vert}–O	180.0	28.0
Fe–C_{hor}–O	180.0	28.0
C_{vert}–Fe–C_{hor}	92.7	100.0
C_{hor}–Fe–C_{hor}	174.6	100.0
**$Fe(CO)_2^+$:**		
Fe–C–O	180.0	28.0
C–Fe–C	180.0	100.0
**$Fe(CO)^+$:**		
Fe–C–O	180.0	28.0

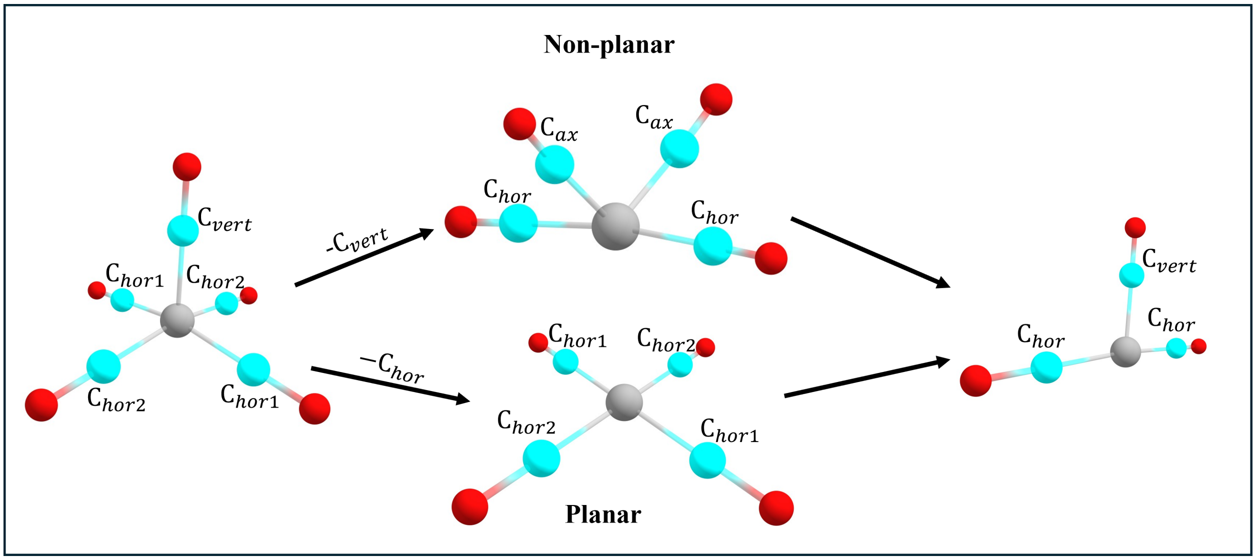

Figure S2: Two $Fe(CO)_5^+$ fragmentation pathways resulting in the formation of planar and non-planar $Fe(CO)_4^+$ structures. The figure also shows the atomic notations forthe $Fe(CO)_5^+$, $Fe(CO)_4^+$, and $Fe(CO)_3^+$ species, which are listed in the interaction parameter tables S3–S5.

Table S5: Partial atomic charges for the singly charged $Fe(CO)_{5-n}^+$ ($n = 0 - 4$) species, employed in the present IDMD simulations. The charge distributions were obtained through the Natural Bond Orbital (NBO) analysis using the Gaussian software package.[1]

Atom	$Fe(CO)_5^+$	$Fe(CO)_4^+$		$Fe(CO)_3^+$	$Fe(CO)_2^+$	$Fe(CO)^+$
		planar	non-planar			
Fe	−0.25438	0.28040	0.2230	0.6181	0.8798	1.0000
C_{vert}	0.42316	–	0.3568	–	–	–
C_{hor2}	0.41240	0.3505	0.3617	0.2965	–	–
C_{hor1}	0.41240	0.3505	–	–	–	–
C_{hor2}	0.41240	0.3505	0.3617	0.2965	0.2241	–
C_{hor1}	0.41240	0.3505	0.3568	0.2965	0.2241	0.1703
O	−0.18101	–	−0.165	–	–	–
O	−0.15934	−0.1706	–	–	–	–
O	−0.15934	−0.1706	−0.165	−0.1692	–	–
O	−0.15934	−0.1706	−0.165	−0.1692	−0.164	–
O	−0.15934	−0.1706	−0.165	−0.1692	−0.164	−0.1703

Non-bonded van der Waals interactions between atoms of the system have been described by means of the Lennard-Jones potential:

$$U_{\mathrm{LJ}}(r_{ij}) = \varepsilon_{ij} \left[\left(\frac{r^{\mathrm{min}}}{r_{ij}} \right)^{12} - 2 \left(\frac{r^{\mathrm{min}}}{r_{ij}} \right)^{6} \right], \tag{S5}$$

where $\varepsilon_{ij} = \sqrt{\varepsilon_i \, \varepsilon_j}$ and $r^{\mathrm{min}} = (r_i^{\mathrm{min}} + r_j^{\mathrm{min}})/2$. The corresponding parameters are listed in Table S6.

Table S6: Parameters of the Lennard-Jones potential, Eq. (S5), describing the van der Waals interaction between atoms of $Fe(CO)_5^+$ and the substrates.

Atom	ε (kcal/mol)	$r^{\mathrm{min}}/2$ (Å)	Ref.
Fe	0.0550	2.27	15
C_{vert}, $C_{\mathrm{hor1,2}}$, C_{ax}, C_{hor}	0.0951	1.95	15
O	0.0957	1.70	15
Au (substrate)	5.2890	1.48	16
C (substrate)	0.0951	1.95	15

Creation of gold and amorphous carbon substrates

As described in the main text, the following substrates have been considered in the present simulations:

- a Au(111) slab with the size of 99.69 Å × 100.73 Å, which contains three atom layers (4200 atoms),
- a Au(100) slab with the size 101.96 Å × 101.96 Å, which contains four atom layers (5000 atoms),
- a polycrystalline gold substrate with the size of 101.96 Å × 101.96 Å, containing 5618 atoms,
- an amorphous carbon (a-C) substrate with the size of 89.17 Å × 89.17 Å, containing 8690 atoms.

The unit cell for an Au(111) slab was constructed using the VESTA software,[17] starting with the fcc unit cell of gold. An orthogonal cell for Au(111) with the lattice parameters of a = 4.985 Å, b = 2.878 Å, and c = 7.049 Å was created. This enabled the construction of a periodic structure in which the Au(111) plane is perpendicular to the z-axis. This unit cell was then used in the crystal generator tool of the MBN Studio software[18] to create an Au(111) substrate of the aforementioned size, consisting of three layers of gold atoms (see Figure 1b in the main text).

The Au(100) substrate was constructed using the built-in crystal generator tool of MBN Studio.[18] A cubic cell with a lattice parameter of a = 4.078 Å was used to create a slab containing 25 unit cells in the in-plane direction and two unit cells in the perpendicular direction.

A similar structure was used as an initial geometry to create a polycrystalline gold substrate. The initially constructed system consisted of 10,000 atoms and comprised 25 unit cells in the in-plane direction and four unit cells in the perpendicular direction. In this

case, the simulation box size was adjusted to match the size of the system in each direction. The system was optimized using the velocity quenching algorithm and thermalized at 300 K for 100 ps. The system was then gradually heated to 1500 K at a rate of 1 K/ps. The molten system was thermalized at 1500 K over 200 ps and then cooled to 300 K at a rate of 1 K/ps. This resulted in the formation of a polycrystalline gold substrate with multiple randomly oriented grains (see the right panel of Figure S3). After that, the system was cut along the z-axis to construct a 10 Å-thick slab containing 5618 atoms. The resulting system corresponded to the initial geometry for the simulations $Fe(CO)_5^+$ adsorption and dynamics.

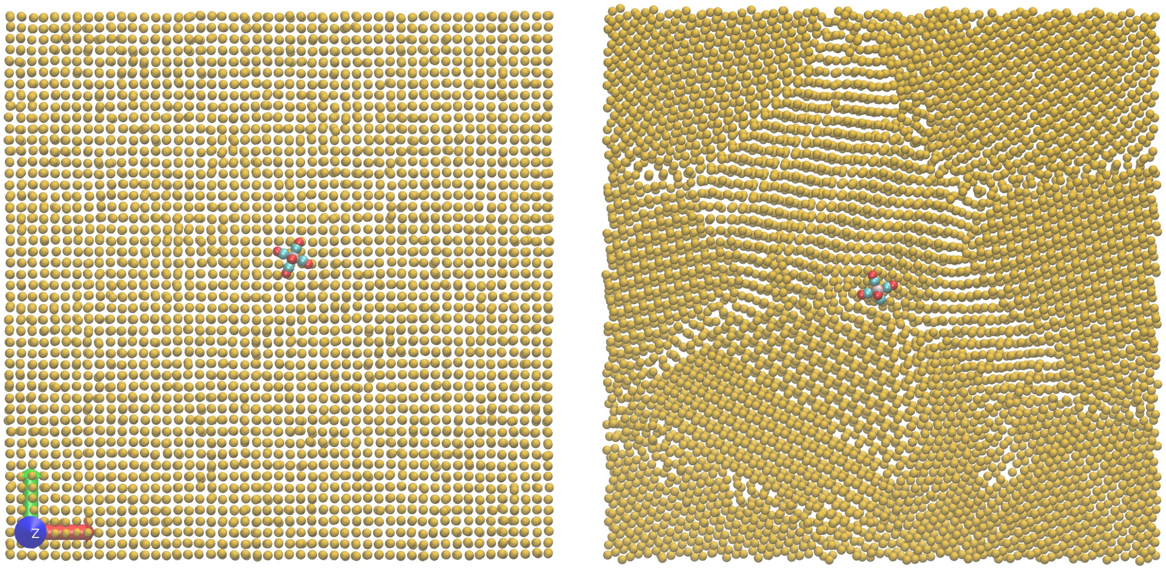

Figure S3: The geometry of the $Fe(CO)_5^+$ on top of a Au(100) substrate (left panel) and a polycrystalline gold substrate (right panel) after thermalization at 300 K. The right panel clearly shows that the created polycrystalline substrate contains multiple randomly oriented nanometer-sized grains. The in-plane size of each substrate is 101.96 Å × 101.96 Å.

Prior to the simulations of $Fe(CO)_5^+$ adsorption, each gold substrate was optimized using the velocity quenching algorithm and thermalized at 300 K for 10 ns. The interactions between gold atoms in the Au(111), Au(100) and polycrystalline gold substrates were described using the many-body Gupta potential[19] with the parameters taken from Ref. 20.

The amorphous carbon (a-C) substrate was constructed as follows. First, a diamond slab with the size of 89.17 Å × 89.17 Å × 19.45 Å (containing 17,500 atoms) was created using

MBN Studio. The interactions between carbon atoms were described using the Brenner potential.[21] This system was annealed by heating to 8,000 K at a rate of 4 K/ps, followed by rapid cooling to 0 K at a rate of 80 K/ps. Next, a part of the system (approximately corresponding to the bottom half) was cut along the z-axis to create a slab containing 8,690 atoms (see Figure 1c in the main text). The resulting system was optimized using the velocity quenching algorithm and thermalized at 300 K for 5 ns to ensure the structural stability of the created substrate.

Accounting for polarization effects induced by the $Fe(CO)_5^+$ ion

To account for the attractive interaction between a molecular ion and a metallic substrate, we introduced an effective atom–surface polarization term of the form $-C_4/r^4$. This choice is physically motivated by the asymptotic interaction between a point charge and a polarizable neutral center,

$$U(r) = -\frac{\alpha q^2}{32\pi^2 \varepsilon_0^2 r^4}, \tag{S6}$$

where α is the polarizability of atoms in a polarizable neutral object and q is the charge. When summed over a semi-infinite substrate with atomic number density n, such a pairwise interaction yields

$$U(z) = -\frac{\pi n C_4}{z}, \tag{S7}$$

which exhibits the same $1/z$ dependence as the exact image interaction of a point charge above an ideal metallic surface,

$$U_{\rm img}(z) = -\frac{q^2}{16\pi\varepsilon_0 z}, \tag{S8}$$

where z is the distance from the charge to the image plane of the conductor. By matching the latter two expressions, one obtains

$$C_4 = \frac{q^2}{16\pi^2\varepsilon_0 n}. \tag{S9}$$

For a multi-center molecular ion described by partial atomic charges q_i, the corresponding atom-specific coefficients are given by

$$C_4(i - \mathrm{Au}) = \frac{q_i^2}{16\pi^2\varepsilon_0 n}, \tag{S10}$$

which reproduce the self-image contribution of each charge center within this approximation.

Using the atomic partial charges for $\mathrm{Fe(CO)}_5^+$ determined from the NBO analysis (see Table S5) and the atomic number density of bulk gold, $n_{\mathrm{Au}} \approx 0.059$ Å^{-3}, we obtained the following coefficients for the effective polarization potential: $C_4(\mathrm{Fe} - \mathrm{Au}) = 1.26$ eV Å^4, $C_4(\mathrm{C} - \mathrm{Au}) = 3.31$ eV Å^4, and $C_4(\mathrm{O} - \mathrm{Au}) = 0.49$ eV Å^4. These parameters reproduce the self-image contribution of each atomic charge center in the long-range limit and provide a computationally efficient approximation to metallic polarization within a nonpolarizable force-field framework.

$Fe(CO)_5^+$ dynamics on Au(111), Au(100) and polycrystalline Au substrates

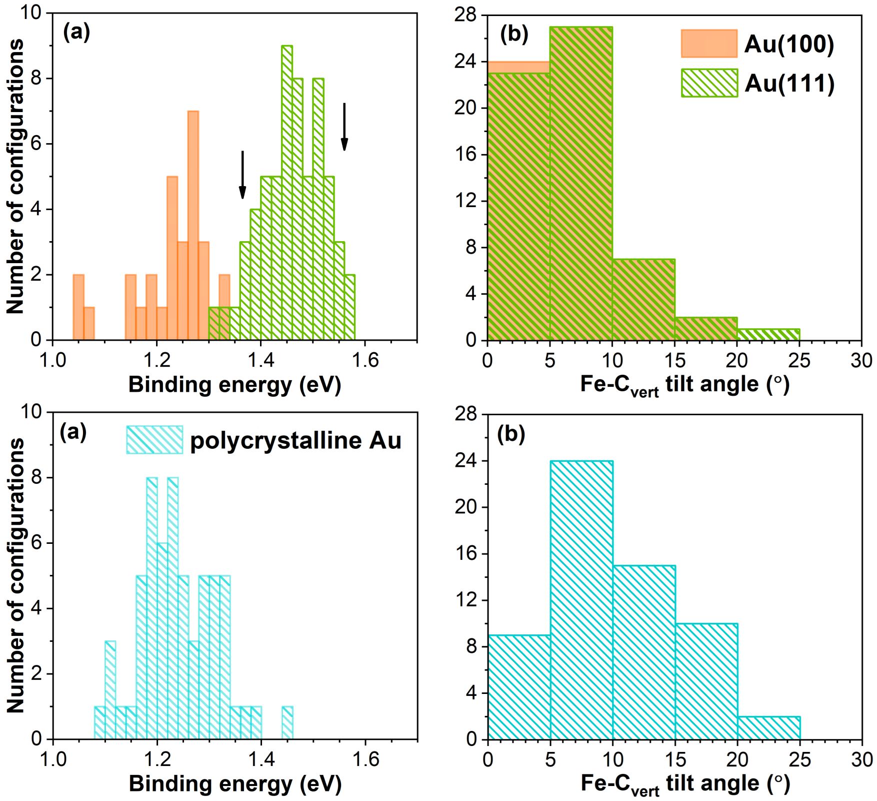

Figure S4: Structural characteristics and energetics of $Fe(CO)_5^+$ adsorption on Au(111), Au(100) and polycrystalline Au substrates. Panels (a,c): The variation of the binding energies of $Fe(CO)_5^+$ on (a) oriented Au(100) and Au(111) substrates, and (c) on the polycrystalline Au substrate. Shaded bars show the number of sampled configurations. Black arrows in panel (a) mark binding energies corresponding to the optimized geometries obtained using the classical interatomic potentials. The bin width is 0.02 eV. Panels (b,d): The variation of the Fe–C_{vert} bond relative to the normal to the substrate. C_{vert} denotes the carbon atom in a nominally vertical CO ligand, which is almost orthogonal to the four other ligands (see Figure 1a in the main text). The bin width is 5°. The presented results are based on 60 independent initial configurations of each system obtained from MD trajectories.

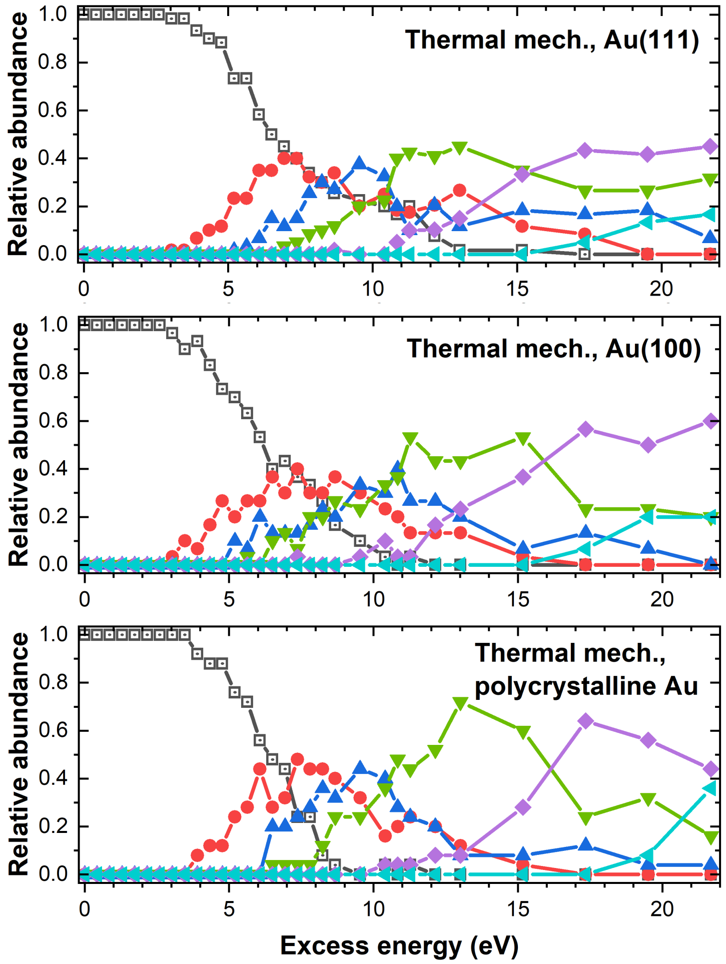

Figure S5: Relative abundances of the parent $Fe(CO)_5^+$ ion and $Fe(CO)_n^+$ ($n = 0 - 4$) fragments as a function of the energy transferred to the parent cation, calculated using IDMD. The top, middle and bottom panels correspond to the crystalline Au(111), Au(100) and the polycrystalline gold substrates, respectively.

Fragment-resolved desorption probability

Figure S7 extends the results presented in Fig. 5 of the main text by providing a breakdown of the contributions of individual fragmentation channels to the overall desorption events.

As described in the main text, 60 independent IDMD simulations were performed for each value of excess energy considered. For illustrative purposes, let us consider the results for the thermal energy deposition mechanism on the Au(111) substrate at an excess energy of 6.1 eV. In 35 trajectories (out of 60), $Fe(CO)_5^+$ remained intact, and no desorption was observed. In

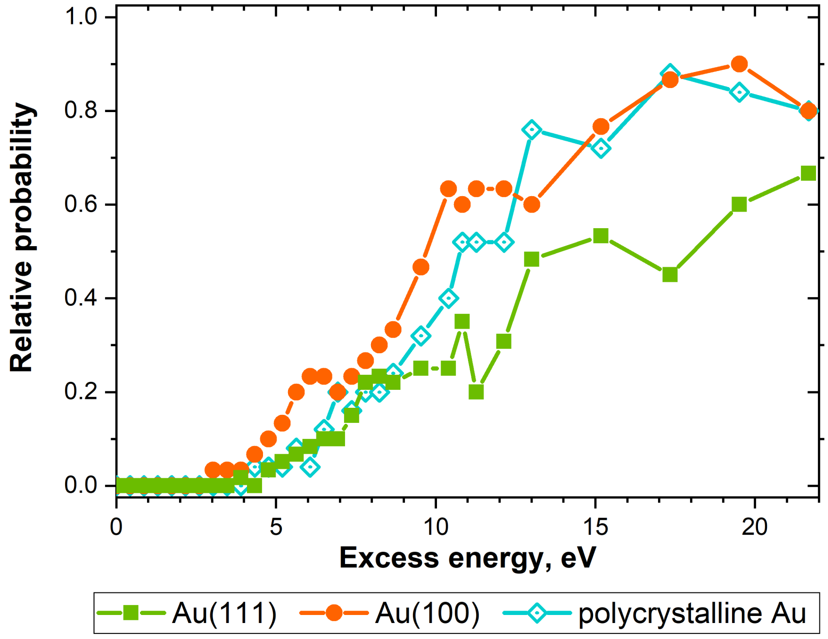

Figure S6: The relative probability of the detachment of Fe-containing species, $Fe(CO)^+_{5-n}$ ($n = 0 - 5$), from the Au(111), Au(100) and the polycrystalline Au substrates as a function of excess energy. The plotted probability is defined as the ratio of the number of desorbed Fe-containing species to the total number of performed IDMD simulations. The results are shown for the thermal energy transfer mechanism.

21 trajectories, fragmentation into $Fe(CO)^+_4$ occurred; in two of these cases, detachment from the substrate was observed (panel (a)), corresponding to approximately 9.5% (panel (c)) of the trajectories in which $Fe(CO)^+_4$ was formed. The remaining four trajectories resulted in the formation of $Fe(CO)^+_3$ fragments, three of which desorbed, corresponding to a desorption probability of 75%. Similarly, panels (b) and (d) of Fig. S7 show the results for the thermal energy deposition mechanism on the a-C substrate.

The species-resolved curves shown in Figure S7 do not span the entire excess energy range because certain fragments are only created above or below specific excess energies. For instance, intact $Fe(CO)^+_5$ is no longer observed at excess energies above approximately 15 eV, whereas desorbed atomic Fe appears only at excess energies exceeding 17 eV.

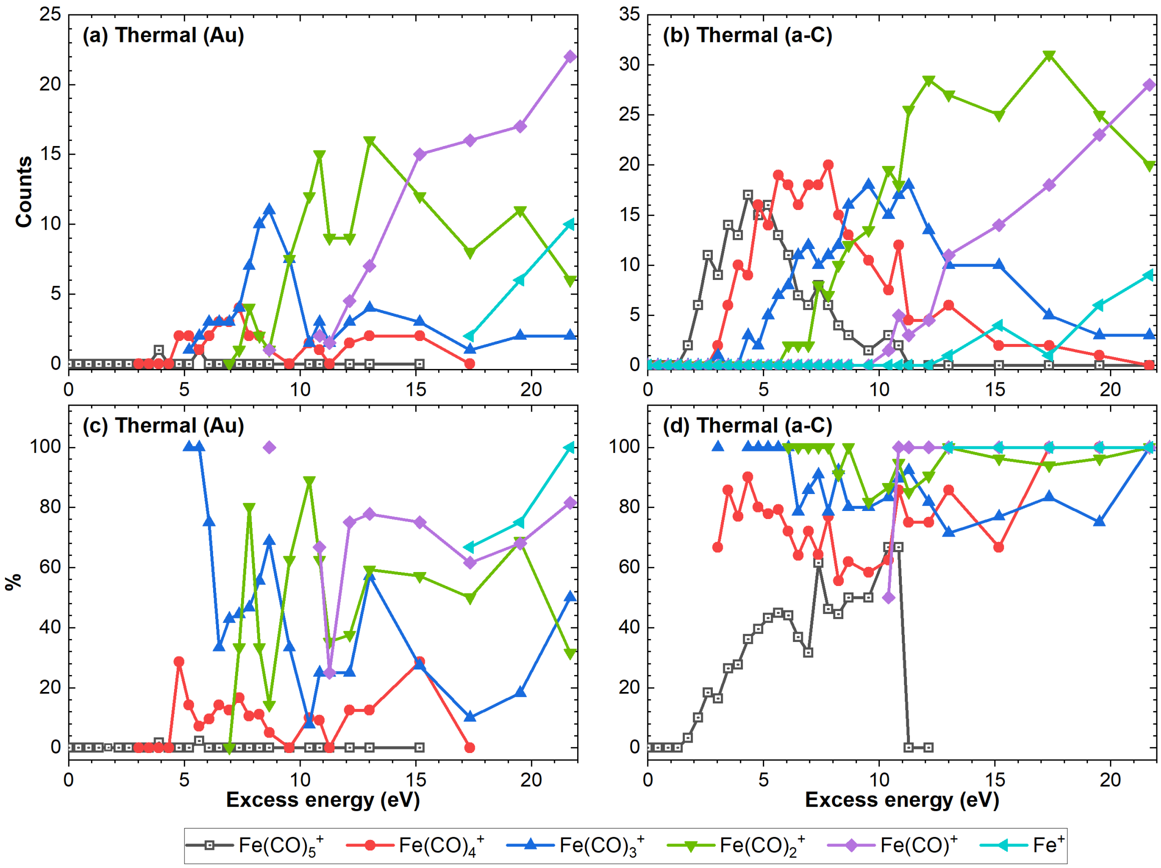

Figure S7: Breakdown of the contributions of Fe-containing fragments, $Fe(CO)^+_{5-n}$ (n = 0–5), to desorption events as a function of excess energy for the thermal energy deposition mechanism. Panels (a) and (c) correspond to the Au(111) substrate, while panels (b) and (d) correspond to the amorphous carbon (a-C) substrate. Panels (a) and (b) show the absolute number of trajectories in which a given Fe-containing fragment desorbs. Panels (c) and (d) show the probability (percentage) of desorption for each fragment, defined as the fraction of trajectories in which a specific fragment desorbs at a given excess energy. The total number of simulations for each excess energy value considered is equal to 60.

References

(1) Frisch, M. J.; Trucks, G. W.; Schlegel, H. B.; Scuseria, G. E.; Robb, M. A.; Cheeseman, J. R.; Scalmani, G.; Barone, V.; Petersson, G. A.; Nakatsuji, H. et al. Gaussian 16 ES64L-G16RevC.01. 2016; Gaussian Inc. Wallingford CT.

(2) Becke, A. D. A New Mixing of Hartree–Fock and Local density-Functional Theories. *J. Chem. Phys.* **1993**, *98*, 1372–1377.

(3) Petersson, G. A.; Bennett, A.; Tensfeldt, T. G.; Al-Laham, M. A.; Shirley, W. A.; Mantzaris, J. A Complete Basis Set Model Chemistry. I. The Total Energies of Closed-Shell Atoms and Hydrides of the First-Row Elements. *J. Chem. Phys.* **1988**, *89*, 2193–2218.

(4) Petersson, G. A.; Al-Laham, M. A. A Complete Basis Set Model Chemistry. II. Open-Shell Systems and the Total Energies of the First-Row Atoms. *J. Chem. Phys.* **1991**, *94*, 6081–6090.

(5) Grimme, S.; Antony, J.; Ehrlich, S.; Krieg, H. A Consistent and Accurate Ab Initio Parametrization of Density Functional Dispersion Correction (DFT-D) for the 94 Elements H-Pu. *J. Chem. Phys.* **2010**, *132*, 154104.

(6) Sushko, G. B.; Solov'yov, I. A.; Verkhovtsev, A. V.; Volkov, S. N.; Solov'yov, A. V. Studying Chemical Reactions in Biological Systems with MBN Explorer: Implementation of Molecular Mechanics with Dynamical Topology. *Eur. Phys. J. D* **2016**, *70*, 12.

(7) Sushko, G. B.; Solov'yov, I. A.; Solov'yov, A. V. Molecular Dynamics for Irradiation Driven Chemistry: Application to the FEBID Process. *Eur. Phys. J. D* **2016**, *70*, 217.

(8) Solov'yov, I. A.; Yakubovich, A. V.; Nikolaev, P. V.; Volkovets, I.; Solov'yov, A. V. MesoBioNano Explorer – A Universal Program for Multiscale Computer Simulations of Complex Molecular Structure and Dynamics. *J. Comput. Chem.* **2012**, *33*, 2412–2439.

(9) Lyshchuk, H.; Verkhovtsev, A. V.; Kočišek, J.; Fedor, J.; Solov'yov, A. V. Release of Neutrals in Electron-Induced Ligand Separation from $MeCpPtMe_3$: Theory Meets Experiment. *J. Phys. Chem. A* **2025**, *129*, 2016–2023.

(10) Verkhovtsev, A. V.; Korol, A. V.; Solov'yov, A. V. Classical Molecular Dynamics Simulations of Fusion and Fragmentation in Fullerene–Fullerene Collisions. *Eur. Phys. J. D* **2017**, *71*, 212.

(11) Friis, I.; Verkhovtsev, A.; Solov'yov, I. A.; Solov'yov, A. V. Modeling the Effect of Ion-induced Shock Waves and DNA Breakage with the Reactive CHARMM Force Field. *J. Comput. Chem.* **2020**, *41*, 2429–2439.

(12) Friis, I.; Verkhovtsev, A. V.; Solov'yov, I. A.; Solov'yov, A. V. Lethal DNA Damage Caused by Ion-induced Shock Waves in Cells. *Phys. Rev. E* **2021**, *104*, 054408.

(13) de Vera, P.; Verkhovtsev, A.; Sushko, G.; Solov'yov, A. V. Reactive molecular dynamics simulations of organometallic compound $W(CO)_6$ fragmentation. *Eur. Phys. J. D* **2019**, *73*, 215.

(14) Andreides, B.; Verkhovtsev, A. V.; Fedor, J.; Solov'yov, A. V. Role of the Molecular Environment in Quenching the Irradiation-Driven Fragmentation of $Fe(CO)_5$: A Reactive Molecular Dynamics Study. *J. Phys. Chem. A* **2023**, *127*, 3757–3767.

(15) Mayo, S. L.; Olafson, B. D.; Goddard, W. A. DREIDING: A Generic Force Field for Molecular Simulations. *J. Phys. Chem.* **1990**, *94*, 8897–8909.

(16) Pohjolainen, E.; Chen, X.; Malola, S.; Groenhof, G.; Häkkinen, H. A Unified AMBER-Compatible Molecular Mechanics Force Field for Thiolate-Protected Gold Nanoclusters. *J. Chem. Theory Comput.* **2016**, *12*, 1342–1350.

(17) Momma, K.; Izumi, F. VESTA 3 for three-dimensional visualization of crystal, volumetric and morphology data. *J. Appl. Crystallogr.* **2011**, *44*, 1272–1276.

(18) Sushko, G. B.; Solov'yov, I. A.; Solov'yov, A. V. Modeling MesoBioNano Systems with MBN Studio Made Easy. *J. Mol. Graph. Model.* **2019**, *88*, 247–260.

(19) Gupta, R. P. Lattice Relaxation at a Metal Surface. *Phys. Rev. B* **1981**, *23*, 6265–6270.

(20) Cleri, F.; Rosato, V. Tight-Binding Potentials for Transition Metals and Alloys. *Phys. Rev. B* **1993**, *48*, 22–33.

(21) Brenner, D. W. Empirical Potential for Hydrocarbons for Use in Simulating the Chemical Vapor Deposition of Diamond Films. *Phys. Rev. B* **1990**, *42*, 9458–9471.
